# The dominance model for heterosis explains culm length genetics in a hybrid sorghum variety

**DOI:** 10.1038/s41598-021-84020-3

**Published:** 2021-02-25

**Authors:** Shumpei Hashimoto, Tatsuro Wake, Haruki Nakamura, Masaki Minamiyama, Satoko Araki-Nakamura, Kozue Ohmae-Shinohara, Eriko Koketsu, Shinnosuke Okamura, Kotaro Miura, Hideo Kawaguchi, Shigemitsu Kasuga, Takashi Sazuka

**Affiliations:** 1 Bioscience and Biotechnology Center, Nagoya University, Nagoya, Japan; 2 Faculty of Bioscience and Biotechnology, Fukui Prefectural University, Eiheiji, Japan; 3 Graduate School of Science, Technology, and Innovation, Kobe University, Kobe, Japan; 4 Faculty of Agriculture, Education and Research Center of Alpine Field Science, Shinshu University, Minamiminowa, Japan

**Keywords:** Genetics, Plant sciences

## Abstract

Heterosis helps increase the biomass of many crops; however, while models for its mechanisms have been proposed, it is not yet fully understood. Here, we use a QTL analysis of the progeny of a high-biomass sorghum F_1_ hybrid to examine heterosis. Five QTLs were identified for culm length and were explained using the dominance model. Five resultant homozygous dominant alleles were used to develop pyramided lines, which produced biomasses like the original F_1_ line. Cloning of one of the uncharacterised genes (*Dw7a*) revealed that it encoded a MYB transcription factor, that was not yet proactively used in modern breeding, suggesting that combining classic *dw1*or *dw3*, and new (*dw7a*) genes is an important breeding strategy. In conclusion, heterosis is explained in this situation by the dominance model and a combination of genes that balance the shortness and early flowering of the parents, to produce F_1_ seed yields.

## Introduction

The phenomenon of heterozygous hybrid plants having superior performance to their parental inbred lines, is known as heterosis. It has been utilized in crop breeding around the world, as it can result in superior seed yields and biomass, and increased resistance to biological and non-biological stresses^1^. Research into heterosis has resulted in different hypotheses for its genetic mechanisms. In brief, four models have been proposed to explain the genetic background of heterosis: dominance^2,3^, overdominance^4–7^, pseudo-overdominance^2,8–10^, and epistasis^11–14^. The dominance model states that heterosis is caused by the complementation of deleterious recessive alleles^2,3^. This model predicts that an inbred line of equal performance to the F_1_ hybrid could be achieved by eliminating all deleterious alleles and accumulating favourable alleles^15^. The overdominance model states that the heterozygous genotype is superior to either of the two homozygous genotypes. The pseudo-overdominance model states that the heterozygous genotypes may exhibit complementation in heterozygous states and act like the overdominance model. In the epistasis model, heterosis is caused by genetic interactions between different loci^13,14^. In addition to these hypotheses, epigenetic regulations such as DNA methylation^16,17^, histone modification^18,19^ and metabolites are also reportedly involved. Heterosis may ultimately be best explained using multiple models.

F_1_ hybrid cultivars of sorghum (*Sorghum bicolour* (L.) Moench) are widely used for forage, as they have large amounts of biomass. In breeding, seed parental lines and/or pollen parental lines, many of which are derived from grain sorghum, have been crossed to evaluate the biomass (or stress resistance) of the F_1_ hybrids. In classic grain sorghum breeding, plant height and maturity (flowering time) are shortened when they are used in the United States, as it is a tropical plant. Four major dwarfing loci (*Dw1-Dw4*) have been used to reduce culm length (CL)^20^. The causal genes for *Dw1, Dw2,* and *Dw3* have been identified as a negative regulator involved in brassinosteroid signal transduction^21^, an AGC protein kinase^22^, and an auxin transport facilitator^23^, respectively. There are other loci (*Ma1-Ma6*) that are used to help control the timing of maturity in sorghum breeding. Among them, *Ma3* and *Ma6* encode *PHYTOCHROME B* (*PhyB*)^24^ and *SbGhd7* (a homolog for the *GRAIN NUMBER*, *PLANT HEIGHT*, and *HEADING DATE 7* gene in rice^25^), respectively.

The F_1_ hybrid variety ‘Tentaka’ is a Japanese cultivar with short parents (~ 120 cm tall), which shows typical heterosis for tallness (~ 4 m tall) and consequently produces a large amount of biomass. We have studied the F_2_ progeny of Tentaka (F_1_) to analyse its genetic basis. Its heterosis for CL could be explained by a combination of five dominant genes using the dominance model. Of the five QTLs identified for CL, an uncharacterised gene was found to encode a MYB transcription factor. These findings will provide new insights to help understand the mechanisms underlying heterosis and develop breeding programs for sorghum and other crops.

## Results

### QTL analysis of flowering date and culm length using the F_2_ population derived from Tentaka

**Figure 1:**
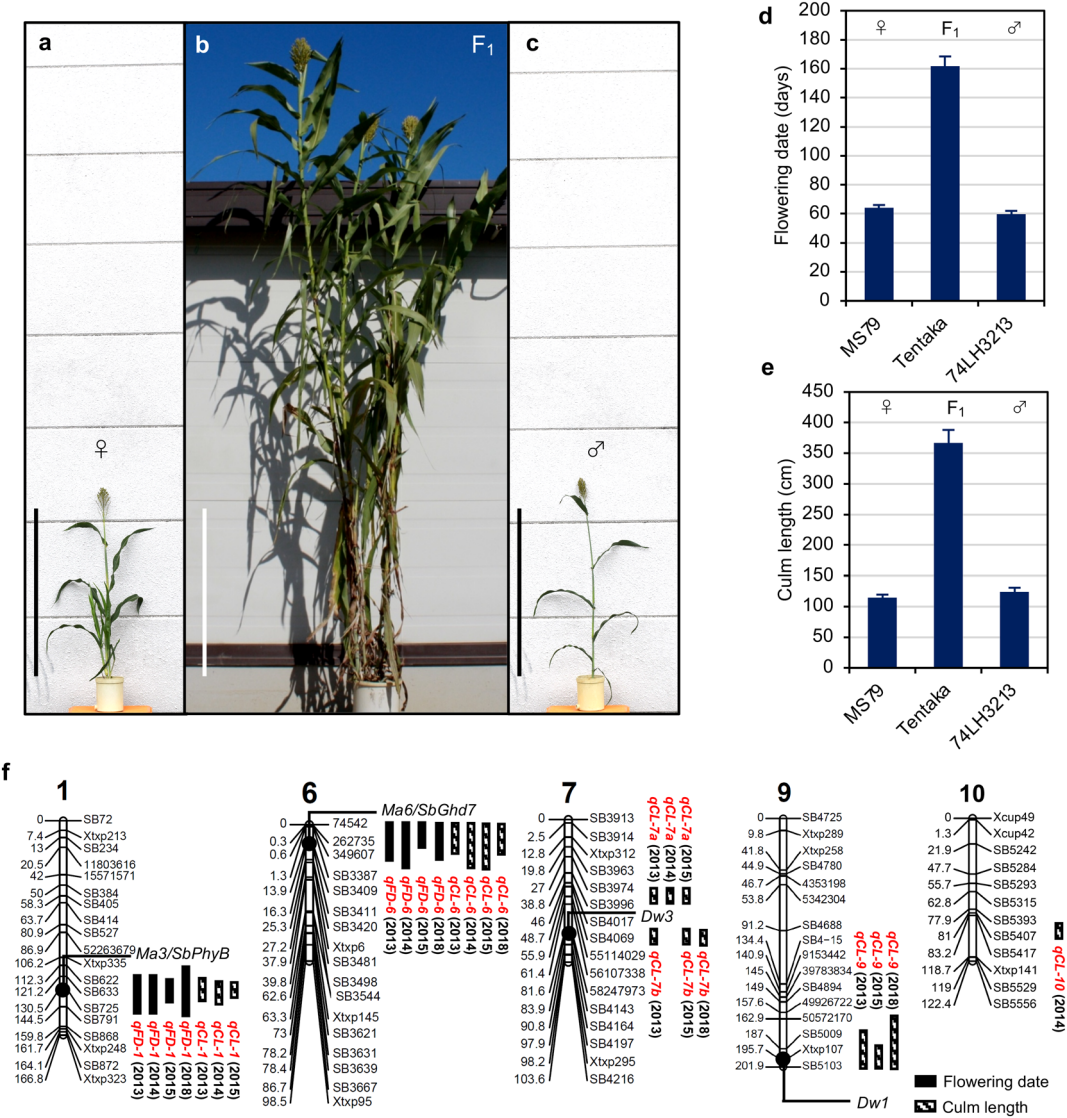
Tentaka is an F_1_ hybrid variety that shows typical heterosis, (**a**–**c**) Plant stature of the F_1_ and its parental lines. (**a**) MS79 (seed parent). (**b**) Tentaka (F_1_); and (**c**) 74LH3213 (pollen parent). Scale bars mean 1 m. (**d**) Flowering date (FD) and (**e**) Culm length (CL) after flowering were investigated in the plants from the parental lines and the F_1_ generation (n ≥ 6, means ± SD). (**f**) An F_2_ population from a cross between MS79 and 74LH3213 was analysed for FD and CL. QTLs for FD and CL with *P* values < 0.05 are presented on the graphical map of the chromosomes, above which the chromosome numbers were mentioned. The positions and names of the DNA markers used for the analysis are indicated on the left and right sides, respectively. The QTL name is indicated in red.

**Figure 2:**
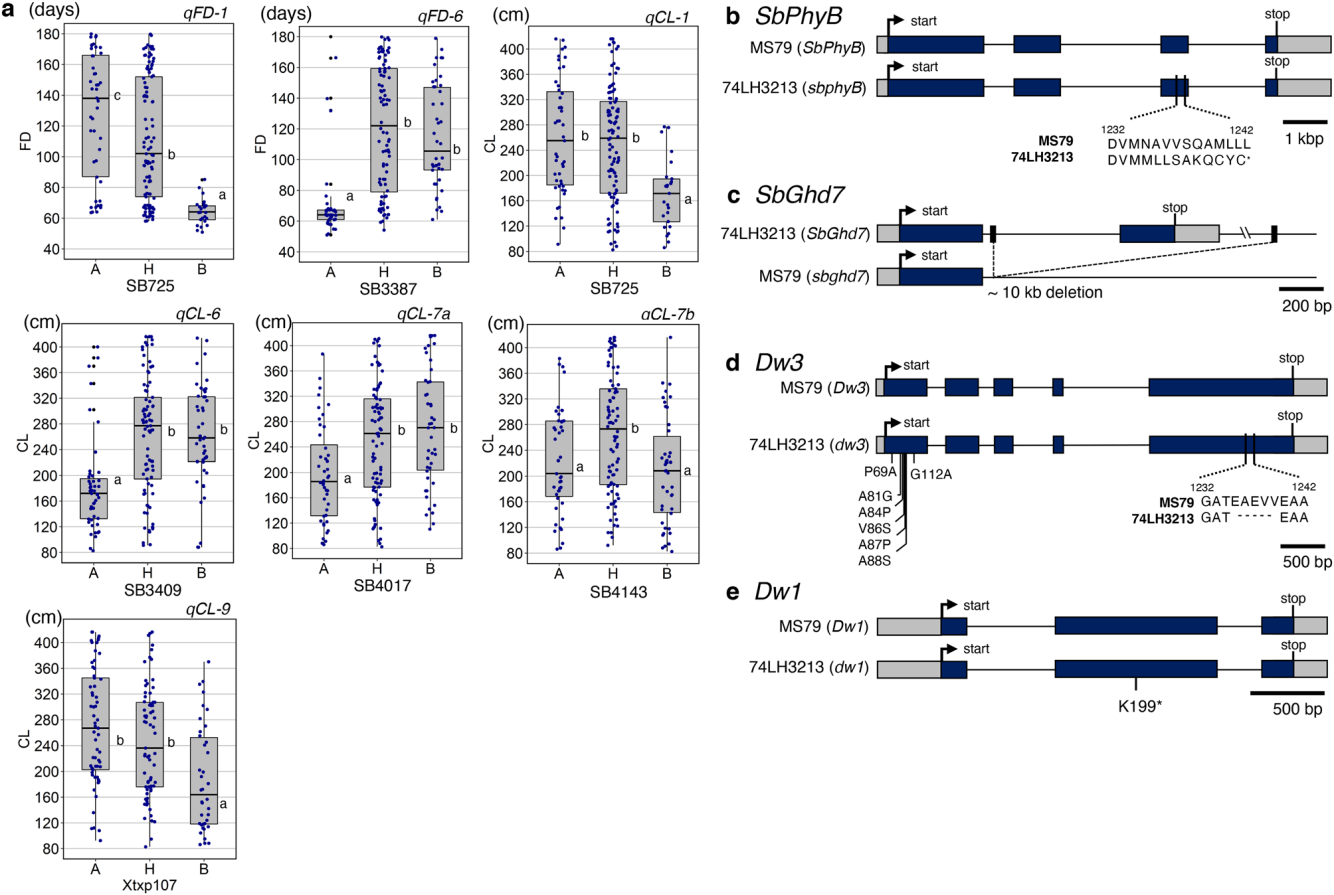
Allelic effects for FD and CL in the F_2_ population, and the candidate corresponding genes for the QTLs. (**a**) Allelic effects of FD and CL in the F_2_ population, as determined from the nearest markers of the identified QTLs. Each plotted point indicates an individual plant in the F_2_ population. A, H, and B on the *x*-axis indicate the homozygous MS79 allele, the heterozygous MS79/74LH3213 allele, and the homozygous 74LH3213 allele, respectively. Only the data from 2015 are shown as a representative sample. (**b**–**e**) Exons and introns are indicated with boxes and black lines, respectively. The dark blue boxes indicate protein coding sequences; the grey boxes indicate untranslated regions (UTR). The alleles in *SbPhyB* (**b**), *SbGhd7*, (**c**), *Dw3* (**d**), and *Dw1* (**e**) are shown.

The Japanese sorghum high biomass F_1_ variety, ‘Tentaka’ (MS79A × 74LH3213), shows intense heterosis (Fig. 1a–c). Two of its parameters, flowering date (FD) and CL, were measured to estimate its biomass. The FD was found to be extremely late, ~ 160 days after sowing (DAS), and it had a long CL (~ 365 cm), when compared to the parental lines that flowered at around 70 DAS and had shorter CL (~ 120 cm) (Fig. 1d,e). QTL analysis was performed for the FD and CL with 155 SSR markers (Fig. 1f, Supplementary Fig. S1). The F_2_ plants were crossed between the MS79A (CMS) and 74LH3213 (pollen parental) lines in 2013 and 2014, and between the MS79B (maintainer) and 74LH3213 lines in 2015 and 2018, to eliminate the linkage of the restorers of fertility (*Rf*) loci. As a result, two significant QTLs for FD were detected on chromosomes 1 and 6 (*qFD-1* and *qFD-6*) in all 4 years (Fig. 1f, Supplementary Fig. S1i–p). *qFD-1* and *qFD-6* showed additive effects from 24.8 to 44.9, and from − 30.1 to − 21.8, respectively, showing that the MS79 allele for *qFD-1* and the 74LH3213 allele for *qFD-6* delayed or prolonged the flowering date (Table 1, Fig. 2a, Supplementary Fig. S2).

**Table 1:** QTLs for flowering date and culm length detected from 2013 to 2015 and in 2018.

Trait	Year	QTL name^a^	Chr	Nearest marker	Position (cM)	LOD	PVE^b^	add^c^	dom^d^	d/a^e^	DPE^f^
Flowering date	2013	*qFD-1*	1	SB725	134.0	11.1	22.8	24.8	18.9	0.76	MS79
		*qFD-6*	6	SB3387	1.0	13.1	28.6	− 25.1	14.5	− 0.58	74LH3213
	2014	*qFD-1*	1	SB725	126.0	11.3	29.5	34.9	22.3	0.64	MS79
		*qFD-6*	6	SB3387	3.0	13.3	32.2	− 28.7	32.0	− 1.12	74LH3213
	2015	*qFD-1*	1	SB725	128.0	10.5	21.2	33.1	16.7	0.50	MS79
		*qFD-6*	6	SB3387	5.0	11.6	18.4	− 21.8	28.8	− 1.32	74LH3213
	2018	*qFD-1*	1	SB633	121.0	13.3	39.7	44.9	11.0	0.24	MS79
		*qFD-6*	6	SB3387	4.0	14.3	38.6	− 30.1	45.0	− 1.49	74LH3213
Culm length	2013	*qCL-1*	1	SB725	133.0	3.0	6.4	20.9	36.8	1.76	MS79
		*qCL-6*	6	SB3387	2.0	7.6	14.8	− 33.6	43.9	− 1.30	74LH3213
		*qCL-7a*	7	SB4069	48.0	2.5	6.4	− 22.1	21.6	− 0.97	74LH34813
		*qCL-7b*	7	SB4164	81.0	2.5	13.8	13.8	35.3	2.55	MS79
		*qCL-9*	9	SB5103	171.0	6.3	10.4	40.9	21.4	0.52	MS79
	2014	*qCL-1*	1	SB725	126.0	4.6	11.9	42.5	31.3	0.74	MS79
		*qCL-6*	6	SB3387	4.0	7.2	19.5	− 46.4	50.6	− 1.09	74LH3413
		*qCL-7a*	7	SB4069	35.0	3.4	8.7	− 16.9	46.3	− 2.73	74LH33713
		*qCL-10*	10	SB5393	76.0	3.1	2.0	24.6	− 43.9	− 1.78	MS79
	2015	*qCL-1*	1	SB725	132.0	4.2	6.9	46.0	38.1	0.83	MS79
		*qCL-6*	6	SB3409	9.0	7.8	15.6	− 42.4	55.0	− 1.30	74LH3913
		*qCL-7a*	7	SB4107	46.5	3.5	3.2	− 37.4	16.2	− 0.43	74LH3213
		*qCL-7b*	7	SB4143	86.0	3.4	7.8	12.0	51.3	4.27	MS79
		*qCL-9*	9	Xtxp107	195.0	4.9	11.5	45.2	22.0	0.49	MS79
	2018	*qCL-6*	6	Chr.6–262735	2.0	9.2	19.1	− 52.1	30.0	− 0.57	74LH3213
		*qCL-7b*	7	SB4216	83.1	5.0	5.4	35.9	29.0	0.81	MS79
		*qCL-9*	9	SB5103	184.0	5.4	8.6	37.5	35.1	0.94	MS79

^a^QTLs were named after each trait and chromosome number. ^b^Percentage of variance explained. ^c^Additive effect of the MS79 allele. ^d^Dominance effect e ratio of dominant to additive effect. ^f^Direction of the phenotypic effect.

For the CL, five notable and reproduceable QTLs were detected on chromosomes 1, 6, 7, and 9 (*qCL-1, qCL-6, qCL-7a, qCL-7b*, and *qCL-9*) over a period of more than three years (Fig. 1f, Supplementary Fig. S2). *qCL-1* and *qCL-6* were found to be the same loci as *qFD-1* and *qFD-6*, respectively, indicating that flowering time (i.e. the number of internodes) influenced CL. The direction of the effect for the *qCL-1* and *qCL-6* was the same as that for the *qFD-1* and *qFD-6*, respectively (Table 1, Fig. 2a, Supplementary Fig. S2). However, it was not excluded that *qFD-6* may influence both the number of internodes and the internode length, like rice *ghd7*^26^. Both QTLs showed additive effects from 20.9 to 46.0, and from − 52.1 to − 33.6, respectively. The alleles of 74LH3213 in *qCL-1* and MS79 in *qCL-6* reduced plant height, like that of *qFD-1* and *qFD-6*, respectively (Table 1, Fig. 2a, Supplementary Fig. 2). A CL-specific QTL, *qCL-9*, whose additive effects ranged from 37.5 to 45.2, and the allele of 74LH3213 were found to reduce plant height (Table 1, Fig. 2a, Supplementary Fig. S2).

### Corresponding genes for each QTL and its alleles

The causal genes for the *qFD* and *qCL* QTLs were investigated by comparing the reported genes involved in FD and CL, between the MS79 and 74LH3213. Based on their map position, we predicted the correspondence of the QTLs to the reported genes as follows: *qFD-1* to *Ma3/SbPhyB*, *qFD-6* to *Ma6/SbGhd7*, *qCL-7b* to *Dw3*, and *qCL-9* to *Dw1*, and found that each parent had null alleles of these genes (Fig. 2b–e). The fact that the pollen parent (74LH3213) had loss-of-function alleles for *ma3/sbphyB*, *dw3*, and *dw1* was consistent with the results that the direction of phenotypic effect (DPE) for all the corresponding QTLs (*qFD-1*, *qCL-7b*, and *qCL-9*, respectively) was for MS79, which has gain-of-function alleles for those genes (Fig. 2b,d–e). In the same way, MS79 has a loss-of-function allele for *ma6/sbghd7* and the DPE for *qFD-6* was for 74LH3213 (Fig. 2c). These results strongly suggest that the QTLs were regulated by the alleles for the causal genes.

In contrast to *qCL-7b/dw3* and *qCL-9/dw1*, *qCL-7a* was not found using the synteny analysis of the known dwarfing genes in other plants to be a candidate gene for the locus. However, the identification of the corresponding gene for *qCL-7a* and its analysis was significant because the gene could combine with the classic dwarfing genes, *dw1* or *dw3*, during F_1_ breeding (see below).

*qCL-7a* is close to *qCL-7b* and showed opposite additive effects and DPE, indicating that the *MS79* allele of *qCL-7a* reduced plant height; however, the MS79 allele of *qCL-7b* increased plant height (Table 1).

### Integration of the five dominant alleles in a pyramided line

**Figure 3:**
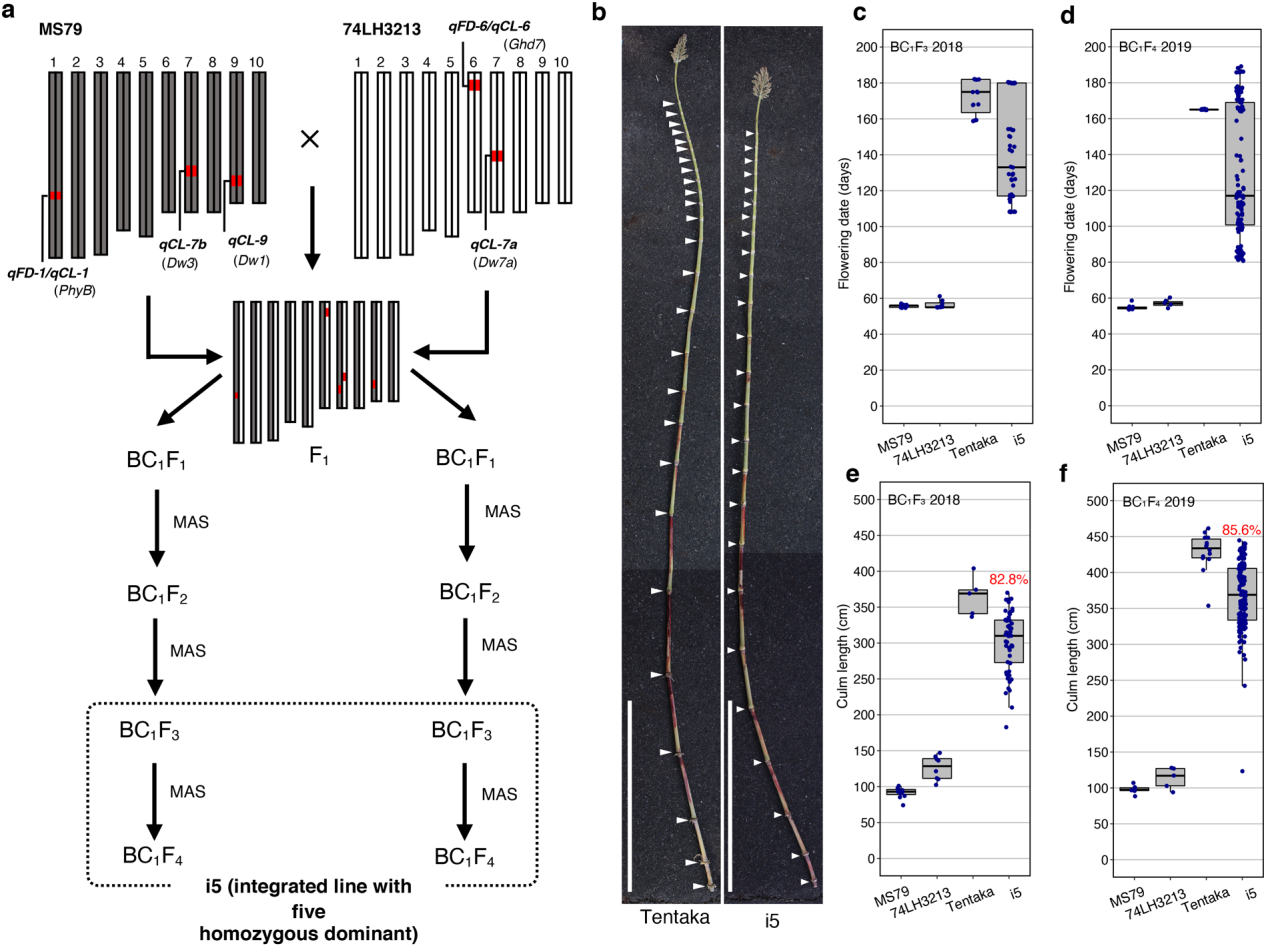
Culm stature of the pyramided lines with five dominant alleles (i5). (**a**) The five dominant alleles (*qFD-1*/*qCL-1*, *qFD-6*/*qCL-6*, *qCL-7a/Dw7a qCL-7b/Dw3*, and *qCL-9/Dw1*) were pyramided by backcrossing and self-pollination, resulting in the development of the i5 lines (BC_1_F_3_ or BC_1_F_4_) (for details, see materials and methods). MAS indicates DNA marker-assisted selection for the pyramiding of the five homozygous dominant alleles. (**b**) Elongation patterns for the internode of an F_1_ hybrid variety ‘Tentaka’ (left) and the pyramided line with five dominant alleles (i5; right). Scale bar, 1 m. (**c**–**f**) Flowering date (**c**,**d**) and culm length (**e**,**f**) of the MS79 (seed parent), 74LH3213 (pollen parent), Tentaka, and i5. Evaluations of the BC_1_F_3_ in 2018 (**c**,**e**) and BC_1_F_4_ in 2019 (**d**,**f**) are shown. The red numbers in panels (**e**) and (**f**) indicate percentages of the i5 when each score of Tentaka is 100.

If heterosis is explained by the dominance model, then it can be fixed by using pyramiding to integrate the dominant alleles into one line. To prove this, we produced a pyramiding line carrying the five homozygous dominant alleles (i5, see Materials and Methods) by backcrossing MS79 × 74LH3213 (F_1_) plants with MS79 or 74LH3213 (BC_1_F_1_), followed by marker-assisted selection and self-pollination (BC_1_F_3_ and BC_1_F_4_) (Fig. 3a–b, Supplementary Fig. S3). As a result, i5 had a delayed flowering time when compared with the parental lines, 142 and 130 days after sowing (DAS) in 2018 and 2019, respectively (Fig. 3c–d). The CL of i5 was 302 cm and 367 cm on average in 2018 and 2019, respectively (Fig. 3e–f); however, for the F_1_ hybrid it was 365 cm and 429 cm in 2018 and 2019, respectively (Fig. 3e–f). These results indicate that the i5 lines mimicked 82.8% to 85.6% of the CL of the F_1_ hybrid, and suggest that the heterosis of the CL at least, is mainly explained by the dominant model.

### Positional cloning of the corresponding qCL-7a gene

**Figure 4:**
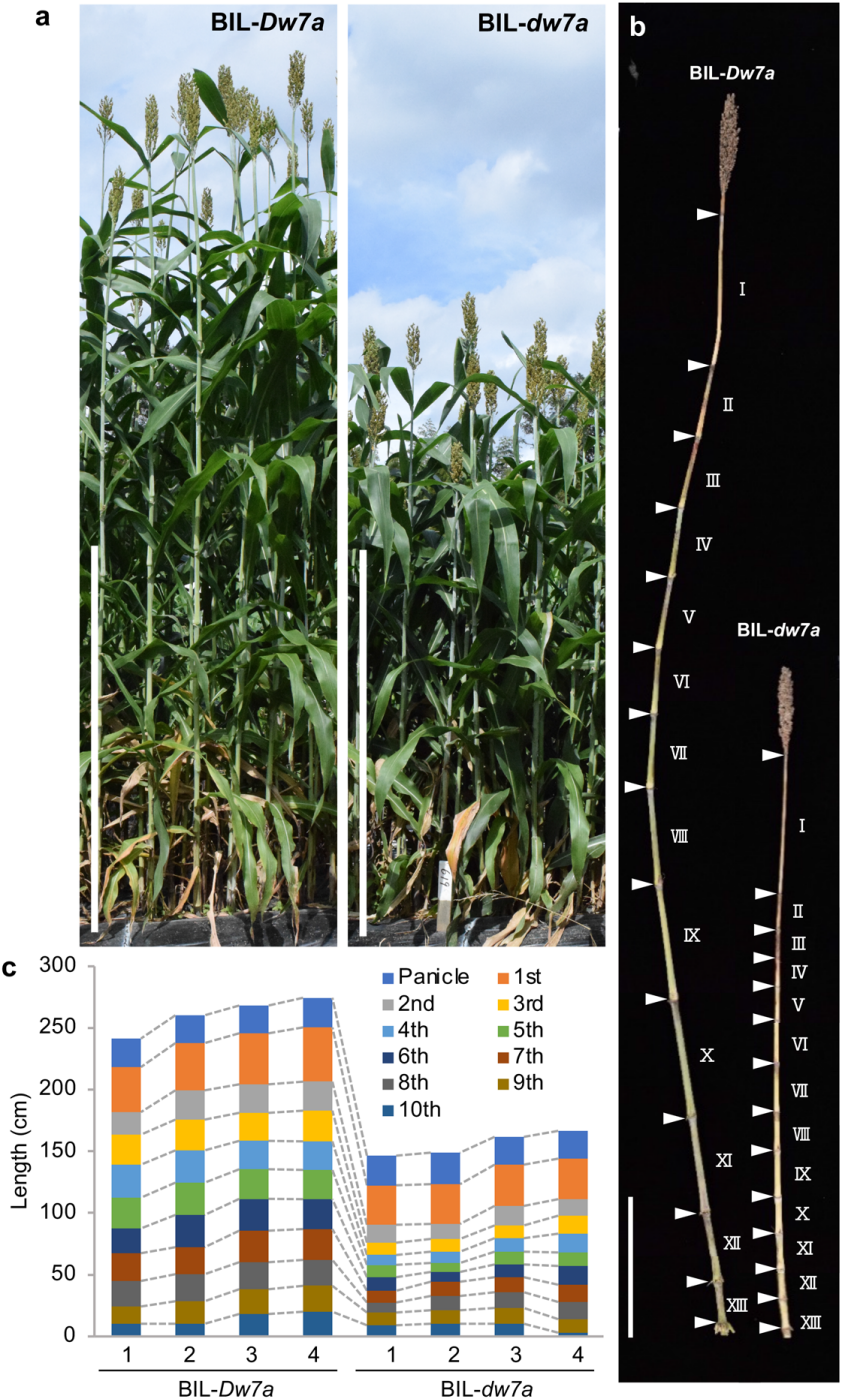
Plant stature and the elongation patterns of the BIL-*Dw7a* and BIL-*dw7a* internodes. (**a**) Plant stature of BIL-*Dw7a* and BIL-*dw7a.* Scale bars = 1 m. (**b**,**c**) Patterns of internode elongation. (**b**) Photograph of the internodes of BIL-*Dw7a* (left) and BIL*-dw7a* (right). The white arrowhead indicates the position of the node and the Roman numerals indicate the internode number. Scale bar = 30 cm. (**c**) The length of each internode of BIL-*Dw7a* and BIL*-dw7a.* Four representative plants are shown from each line.

**Figure 5:**
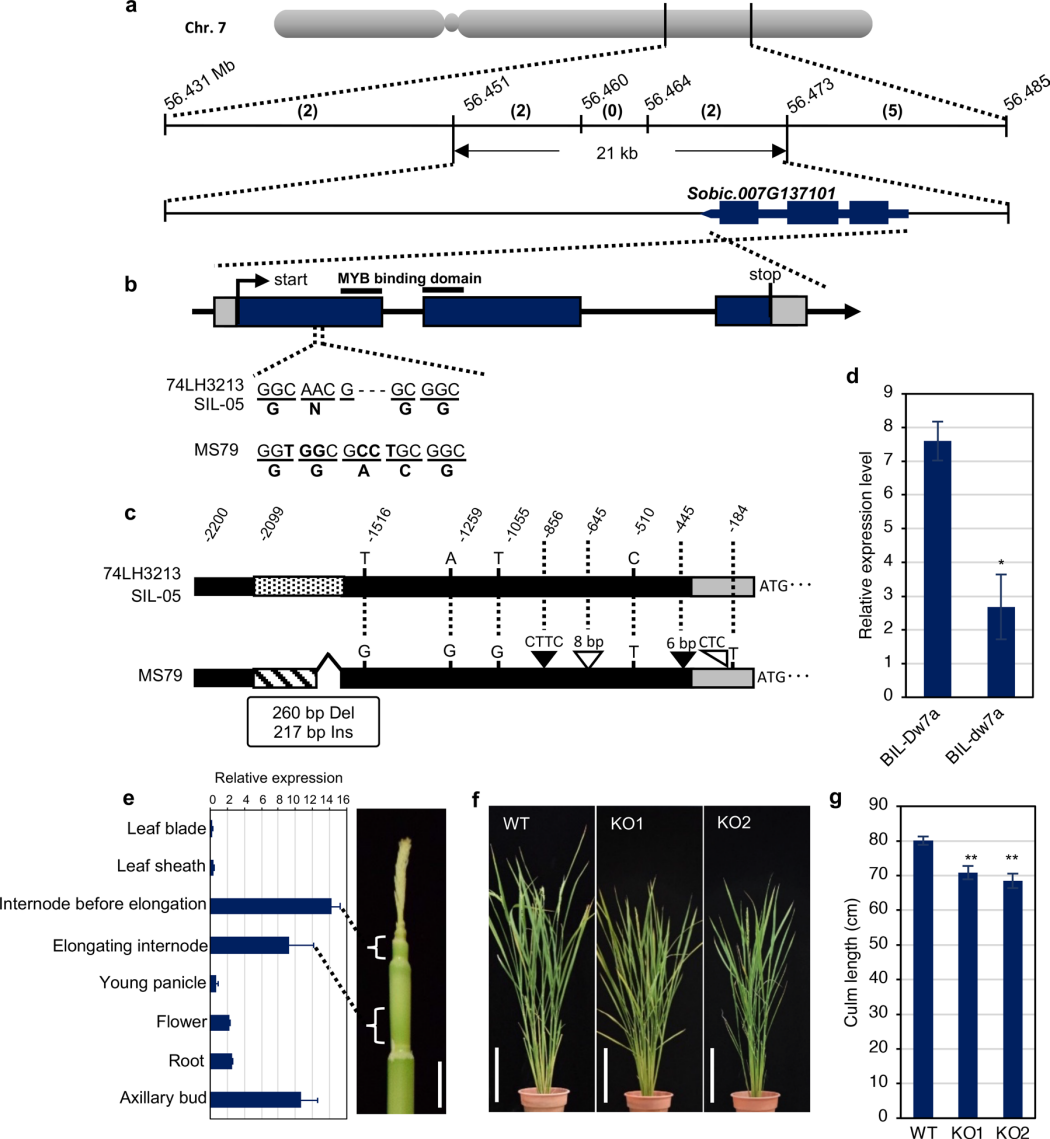
Positional cloning of *Dw7a* and phenotypes of the CRISPR/Cas9 mediated knock-out lines of *OsDw7a* in rice. (**a**) Physical position of *Dw7a* (corresponding gene of *qCL-7a*). The uppermost grey bar indicates chromosome 7. On the upper horizontal line, the vertical lines indicate the physical positions of the DNA markers (Mb), and the number of recombinants is shown in parentheses between the markers. The bottom horizontal line represents the candidate region of 21 kb. Exons are represented as black boxes. (**b**) Gene structure of *Dw7a* (Sobic.007G137101). Exons are represented as dark blue boxes and the 3ʹ or 5ʹ-untranslated regions (UTR) are indicated with grey boxes. The bold lines indicate the R2R3 MYB binding domain. (**c**) Four SNPs (indicated by nucleotides), three In/Dels (black/white triangles, respectively), genomic fragment substitution (260 bp to 217 bp; striped bar) in the promoter region (dotted and striped bares), and one SNP and one deletion in the 5ʹ-UTR region (grey bar) are shown. (**d**) The expression levels of the *Dw7a* in the BIL-*Dw7a* and BIL-*dw7a* (two-tailed Student’s *t*-test, *P < 0.05, n = 3). (**e**) The expression levels of *Dw7a* (in cultivar SIL-05) in different organs. Scale bar in the photograph = 2 cm. The positions of the two internodes ‘before elongation’ and ‘elongating’ are shown in the photograph. (**f**) Phenotype at the heading stage of the WT and two knockout lines. Scale bar = 20 cm. (**g**) Length of each internode of the WT and knockout lines (two-tailed Student’s *t*-test, **P < 0.01, n = 6).

A causal gene for *qCL-7a* that was called *Dw7a*, was cloned by crossing a Japanese sweet sorghum variety SIL-05 (*Dw1 Dw3 Dw7a ghd7*) and MS79B (*Dw1 Dw3 dw7a ghd7*), which showed clear differences in their CLs. Segregating lines (BC_3_F_6_ or BC_3_F_7_) for *Dw7a* were developed and subjected to analysis (Fig. 4a–c, Supplementary Fig. S4a–c, see Materials and Methods). The candidate region was narrowed down to 21 kb between the markers located at 56.45 Mb and 56.47 Mb (Fig. 5a), where only one annotated gene (Sobic.007G137101) was predicted (Fig. 5a–b). The gene encodes an R2R3 type MYB transcription factor with an FxDFL motif^27^, and in MS79B it contained a 3 bp insertion and 3 bp substitution (NG^SIL-05^ to GAC^MS79^) in the first exon in comparison to SIL-05, whereas there were also some nucleotide differences 2.2 kb upstream (Fig. 5c). Phylogenetic analysis was carried out using the orthologues of this gene in monocots and dicots (Supplementary Fig. S4d). Interestingly, the results showed that the clade of *Poaceae* showed a distant genetic relationship from *Ananas* or *Musa*, and from a wider perspective, also from dicots, suggesting the possibility of a specific function for the DW7a of *Poaceae*.

### Expression of the Dw7a and dw7a plant phenotypes

qRT-PCR analysis showed that the expression of *Dw7a* was almost three times higher than that of *dw7a* in the internodes (Fig. 5d), where *Dw7a* is actively expressed relative to other organs (Fig. 5e). To support the hypothesis that Dw7a controls CL, we produced rice knock-out plants defective in its homologues, as a reliable knockout system has been established for rice, but not for sorghum. The structural comparison and syntenic relationship clearly demonstrated that rice LOC_Os08g33800 (named OsDW7a) was the counterpart of the sorghum DW7a and Sobic.007G137101 (Supplementary Fig. S4d). The rice knock-out lines had shorter internodes than the wild-type (Fig. 5f–g, Supplementary Fig. S4e), which mimicked the dwarf phenotype of the backcross inbred line (BIL)-*dw7a* of sorghum. Furthermore, there were no other pleiotropic effects of *dw7a* in sorghum, except for stem elongation*,* as was seen in *BIL-dw7a* (Supplementary Fig. S4f–o), which is an essential characteristic of dwarf genes for practical breeding, and was also seen in the rice knockout plants.

### Haplotype analysis of dw7a

**Figure 6:**
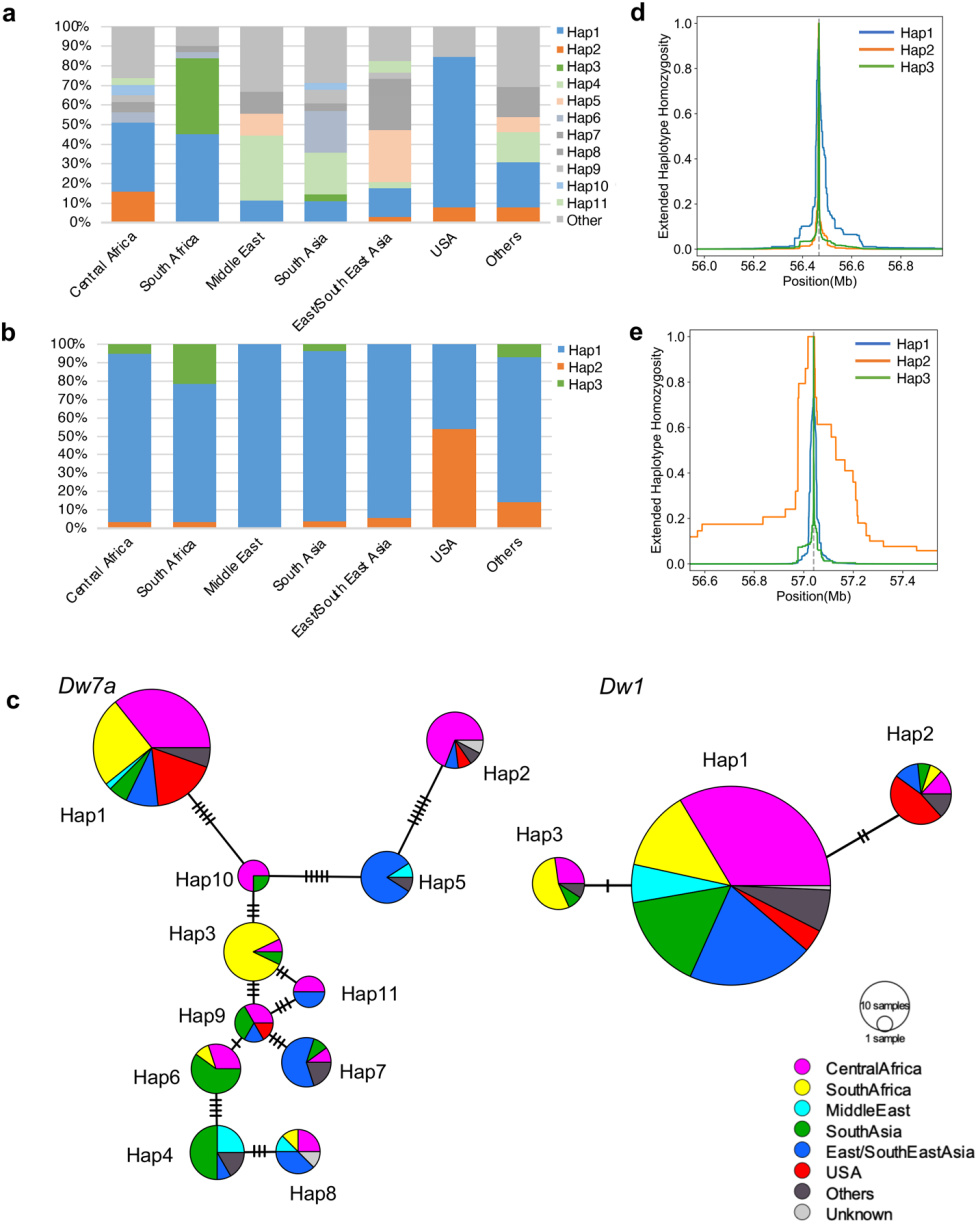
Haplotype analyses of *Dw7a* and *Dw1*. (**a**,**b**) Regional distribution of each haplotype of the *Dw7a* (**a**) and *Dw1* (**b**). (**c**) Haplotype network of *Dw7a* and *Dw1* using 187 accessions. Hatch marks between each haplotype indicate the mutational step. In *Dw7a*, Hap1 to Hap11 were used for analysis (147 accessions). (**d**,**e**) Extended haplotype homozygosity (EHH) decay of *Dw7a* (**d**) and *Dw1* (**e**). Hap1 (blue), Hap2 (orange), and Hap3 (green) are shown. Dotted lines indicate the positions of *Dw7a* (**d**) and *Dw1* (**e**).

To study the distribution of the *dw7a* allele, a haplotype analysis was carried out using whole genome sequences from 187 accessions, that included part of the ICRSAT mini core collection^28^, the NARO panel^29^ and the available lines in our lab (see Materials and Methods). The *Dw7a* alleles were categorised into 11 haplotypes (allele frequency > 1%) with 8 SNPs and 6 indels in its genome region (Fig. 6a, Supplementary Fig. S5a). In contrast, *Dw1*, which is widely used in US modern breeding, was classified into 3 haplotypes, all of which have previously been reported^30^ (Fig. 6b). Haplotype network analysis found that Hap1 of *Dw7a* could be ancestral, as it was the most frequent, was wide spread, and Hap10, Hap5, and Hap2 (the haplotypes of the short culm in this study) were derived from it, sequentially (Fig. 6c). In *Dw1*, Hap1 was also found to be ancestral, whereas Hap2 (the haplotype of short culm, and of the loss-of-function shown in the previous study^30^) and Hap3 were directly derived from Hap1. We performed extended haplotype homozygosity (EHH)^31^ analysis and compared the EHH decay between the gain- and loss-of-function haplotypes of *Dw7a* and *Dw1*. For *Dw1*, Hap1, and Hap3 (gain-of-function) occurred rapidly, relative to Hap2 (loss-of-function) (Fig. 6e), but no significant differences were observed in the case of *Dw7a* (Fig. 6e). The integrated haplotype score (iHS)^32^ analysis statistically confirmed the above prediction (Supplementary Fig. S5b,c). These results indicate that the loss-of-function haplotype, Hap2 of *Dw7a*, had not been selected by any processes previously, such as breeding, bottlenecks, or evolution; in contrast to Hap2 of *Dw1,* which had been selected by breeding (Fig. 6d–e; see discussion).

It should be noted that haplotypes MS79 and 74LH3213 belong to the Hap2 and Hap1 groups, respectively, and central Africa and South Africa are their major sites of origin (Fig. 6c, Supplementary Table S1).

## Discussion

Increasing yields by using F_1_ hybrids, is a fundamental aspect of global agriculture; however, the mechanisms of heterosis, contributing genes, or combinations thereof, have not yet been elucidated. To improve our understanding, we have conducted a genetic analysis of the sorghum variety ‘Tentaka’, a Japanese F_1_ hybrid, which shows typical and intense heterosis.

The genetic and molecular biological analyses revealed that 74LH3213 possesses three mutations: *qFD-1/sbphyB* for early flowering, and *qCL-7b/dw3* and *qCL-9/dw1* to reduce internode length, and all three reduce CL, whereas MS79 possesses two mutations, *qFD-6/sbghd7* for early flowering and CL reduction, and *qCL-7a/dw7a* for reducing CL alone (Table 2, Supplementary Fig. S6). The loss-of-function and gain-of-function allele combinations for these genes, strongly suggests that the heterosis of Tentaka follows the dominance model. This prediction was confirmed by the pyramided line i5, which carries the five dominant alleles and achieved approximately 82.8–85.6% of the heterosis of the CL when compared with that of Tentaka. The reasons for the remaining 14.4–17.2% difference in heterosis are currently unclear but may be due to contributions from the remaining weak QTLs. For example, the *SbPRR37/Ma1* regulator of flowering time is a possible candidate^33^, because the alleles of both parents are different (Supplementary Fig. S7), but significant QTLs were not detected. The weak contributions of over dominant genes, such as *qCL-7b/Dw3* (d/a = 2.55 in 2013, and 4.27 in 2015), were observed in this study (Table 1).

**Table 2:** Summary of the detected QTLs for flowering date and culm length and the candidate corresponding genes.

QTL name	Trait	Corresponding gene	Genotype			
			MS79	74LH3213	Tentaka	i5
*qFD-1*	Flowering date	*PhyB*	*PhyB PhyB*	*phyB phyB*	*PhyB phyB*	*PhyB PhyB*
*qFD-6*		*Ghd7*	*ghd7 ghd7*	*Ghd7 Ghd7*	*ghd7 Ghd7*	*Ghd7 Ghd7*
*qCL-6*	Culm length	*Ghd7*	*ghd7 ghd7*	*Ghd7 Ghd7*	*ghd7 Ghd7*	*Ghd7 Ghd7*
*qCL-7a*		Dw7a*	*dw7a dw7a*	*Dw7a Dw7a*	*dw7a Dw7a*	*Dw7a Dw7a*
*qCL-7b*		*Dw3*	*Dw3 Dw3*	*dw3 dw3*	*Dw3 dw3*	*Dw3 Dw3*
*qCL-9*		*Dw1*	*Dw1 Dw1*	*dw1 dw1*	*Dw1 dw1*	*Dw1 Dw1*

*Identified in this study.

Genetic studies to elucidate heterosis have been reported for *Poaceae*. For example, Huang et al. generated, sequenced, and recorded the phenotypes of 10,074 F_2_ lines from 17 representative hybrid rice crosses^34^. As a result, they observed several significant dominance effects for heterosis, concerning traits related to grain yield. Among them, the candidate genes for the 23 QTLs were identified. Interestingly, most of the QTLs showed positive dominance effects in the heterozygous state.

Another example for the model was reported by Li et al.^8^, in which two significant QTLs for plant height, *Dw3* and *qHT7.1*, showed a repulsion linkage in the two parental cross populations^8,35^. This is reported as the pseudo‐overdominance model; however, except for the loci of the QTLs showing tight genetic linkage, the model was considered a dominance model in the broad sense. Although *Dw3* only affects the area below the flag leaf, *qHT7.1* affects both the upper and lower parts of the plant^8^, and the dominance model does not seem to contradict these results. Together with the results of the above studies, the dominance model may explain the heterosis for these two traits in some cereals, and could be a candidate model to utilise with other crops*.* Surprisingly, however, the focus of this investigation was relatively simple and clearly resolved; as only 5 genes contributed to the dominance model, while dozens of QTLs were detected in rice.

This could be explained by the origin and history of the parental lines, as both parental lines of Tentaka were originally considered grain sorghum. In breeding grain sorghum, two traits, early flowering, and low plant height, are significant for stable seed production. Early flowering contributes to a shorter field culture period, which reduces the damage from weather (low temperature, rain, etc.), diseases, and insects, and low plant height aids in mechanical harvesting. From this point of view, it is natural that both parental lines contain one early flowering gene and one or two dwarfing genes, which are necessary for grain sorghum in its original pedigree. In addition, they must not be the same genes used between the pollen parental line and the seed parental line, because the F_1_ plants would flower even earlier or become shorter in height, and consequently, have reduced biomass. Classic F_1_ breeding has been carried out using random crossing tests between the CMS and pollen lines, resulting in multiple combinations over multiple years. Effort is required by a breeder to establish the right combination of parents to produce the F_1_ varieties demonstrating heterosis, while obviating the issue of homozygosity in which early flowering and dwarfing genes must be carried out in both parental lines. As described above, the reason that the four genes, *ma3*/*phyB, ma6/ghd7, dw1,* and *dw3*, are present in the parental lines, is that they are frequently used in modern breeding. For example, Hap2 in *dw1* was a spontaneous mutation in 1905, and many cultivars now carry the *dw1* mutation for lodging resistance and to improve mechanised harvesting. This history reflected the results of the high EHH decay in haploid analysis (Fig. 6e, Supplementary Fig. S5b). However, *dw7a* is different because it has not been characterised and is not yet used proactively in modern breeding, as shown by the low EHH decay (Fig. 6d, Supplementary Fig. S5a). These results suggest not only the importance of *dw7a*, but also the necessity to identify new genes that do not overlap with the classic dwarfing genes for breeding F_1_ hybrids in the future.

## Materials and methods

### Experimental design

All field experiments were carried out at the Togo Field Science and Education Center of Nagoya University (Aichi, Japan). An F_2_ population was derived from the sorghum hybrid cultivar ‘Tentaka’ (MS79 × 74LH3213), whose parents were grown in 2013, 2014, 2015, and 2018, and the BIL population (see below) was grown in 2017 and 2018. These seeds were sown in a nursery bed in a greenhouse, and 4-week-old seedlings were transplanted to the field with 15 cm spacing between each plant, in two rows (30 cm spacing) per hill (100 cm in width), and each furrow was 80 cm wide.

### QTL analysis

The F_2_ populations (n = 173 in 2013, 171 in 2014, 182 in 2015, and 144 in 2018) that were derived from crosses between MS79 and 74LH3213, were used for QTL analysis. The CL was measured from the ground to the panicle node, and the flowering date was set as the number of days from sowing to flowering on the main panicle. Linkage analyses and QTL identifications were performed using R/qtl software v1.46^36^ based on genotypic data, while recombination frequencies were converted to genetic distances in centimorgans (cM), using the Kosambi function^37^. The primers used in this study are listed in Supplementary Table S2. The threshold for each dataset was based on a permutation test (1,000 permutations) and *P* = 0.05. The box plots for allelic effects were plotted using R studio v1.2.5033 package ggplot2 (http://ggplot2.tidyverse.org)^38^.

### Positional cloning of Dw7a

For positional cloning of *Dw7a*, segregating populations of MS79 × SIL-05 (BC_3_F_6_ or BC_3_F_7_), in which the *Dw7a* locus was heterozygous (*Dw7a dw7a*) were used. A total of 2,833 plants were studied in 2017 and 2018. For the phenotype analysis, the MS79 × SIL-05 (BC_3_F_9_) lines that carried the locus and were homozygous dominant (*Dw7a Dw7a*; *BIL-Dw7a*) and homozygous recessive (*dw7a dw7a*; *BIL-dw7a*), were developed and studied in 2019.

### Genotyping

Genomic DNA was isolated from the leaves of ~ 6-week-old plants in the field using a cetyltrimethylammonium bromide (CTAB) extraction method^39^ with some modifications. In brief, leaf samples were ground using a MultiBead Shocker (Yasui Kikai, Osaka, Japan) in 2 × CTAB extraction buffer [100 mM TrisHCl (pH 8.0), 50 mM EDTA, 1.4 M NaCl, 2% CTAB, 1% PVP]. After incubation at 60 °C for 30 min, an equal volume of chloroform was added. After centrifugation at 15,000 rpm for 5 min, the supernatant was recovered, and an equal volume of isopropyl alcohol was added. The sample was recovered by centrifugation at 15,000 rpm for 5 min, and the pellet was washed with 70% ethanol. The DNA was dried and dissolved in 1 × Tris/EDTA (TE) solution (10 mM Tris–HCl [pH 8.0], 1 mM EDTA [pH 8.0]). The purified DNA samples were then used for genotyping using polymerase chain reaction (PCR). SSR markers, as reported by Yonemaru et al.^40^ were screened, and the markers showing polymorphisms between the two parents were selected. DNA segments were amplified by PCR with the following program: 95 °C 1 min, (95 °C 30 s, 55 °C 30 s, 72 °C 30 s) × 30 cycles, 72 °C 7 min. PCR products were analysed by gel electrophoresis.

### Phylogenetic analysis

The amino acid sequences of the DW7a orthologs were obtained using the Ensembl Plants database (https://plants.ensembl.org/), Phytozome (https://phytozome.jgi.doe.gov/pz/.

portal.html), and NCBI (https://www.ncbi.nlm.nih.gov). Phylogenetic tree analysis was carried out using the neighbour-joining method, based on the JTT model^41^ using MEGA X^42^. Bootstrap values were obtained from 1000 replicates.

### Plasmid construction and transformation experiments

For knockouts of the *OsDw7a*, synthetic genomic RNA (gRNA) was inserted into CRISPR/Cas9 plasmids, as previously described by Endo et al.^43^. The 20-nt oligonucleotides targeting the *OsDw7a* (LOC_Os08g33800) sequences were annealed and cloned into the BbsI recognition sites of pU6gRNA. Then, the gRNA expression cassette in the pU6gRNA vector was ligated into a gRNA/Cas9-expressing binary vector (pZDgRNA_Cas9ver.2_HPT), using the AscI and PacI sites. pZDgRNA_Cas9ver.2_HPT vectors were introduced into calli (*Oryza sativa* L. ‘Nipponbare’) through *Agrobacterium tumefaciens* (EHA105) mediated transformation, according to Ozawa^44^. In the T_1_ generation, the knockout lines that did not contain the Cas9 gene were selected and transgenic plants of the T_2_ generation were used for the analysis.

### Gene expression analysis

Total RNA was isolated from the plant samples using the RNeasy Plant Mini Kit (Qiagen, Hilden, Japan), according to the manufacturer’s guidelines. A total of 500 ng of total RNA was used to synthesise the first strand cDNA, using an Omniscript reverse transcription (RT) kit (Qiagen), according to the manufacturer’s instructions. Quantitative RT-PCR (qRT-PCR) was conducted using KOD SYBR qPCR Mix (TOYOBO, Osaka, Japan) and a real-time thermal cycler (Bio-Rad Laboratories, Hercules, CA, USA). The sorghum ubiquitin gene (Sobic.001G311100; *SbUbi*) was used as the internal reference for all analyses.

### QTL pyramiding

The MS79 × 74LH3213 (BC_1_F_1_) plants, were a cross between MS79 × 74LH3213 (F_1_) and MS79 or 74LH3213, and were genotyped using the nearest SSR markers for the five loci (*qFD-1/qCL-1*, *qFD-6/qCL-6*, *qCL-7a*, *qCL-7b*, and *qCL-9*), and plants were selected that were homozygous dominant or heterozygous for these loci. BC_1_F_2_ or BC_1_F_3_ plants were selected again by genotyping, and the lines BC_1_F_3_ and BC_1_F_4_ were homozygous dominant for all five loci (i5; integrated line with five homozygous dominant). The i5 lines were grown in the field and their agronomic traits were evaluated in 2018 and 2019. The box plots were plotted using R studio v1.2.5033 using package ggplot2 (https://ggplot2.tidyverse.org)^38^.

### Haplotype analysis

A total of 187 accessions of *Sorghum bicolor* (L.) Moench, taken from the ICRISAT (International Crops Research Institute for the Semi-Arid Tropics) mini core collection^28^, the sorghum diversity research set of NARO (National Agriculture and Food Research Organization)^29^, and the available lines in our lab, were used for this study (Supplementary Table S3). A part of the accessions was sequenced using an Illumina Hiseq X Ten sequencer (Illumina, San Diego, CA, USA) by pair-end sequencing to obtain the SNPs around the *Dw7a* locus, and the data for the whole genome sequences of the remaining accessions were obtained from Prof. Tsutsumi (University of Tokyo) (Supplementary Table S3). In SNPs calling, after adapters were trimmed and low-quality reads were filtered, the clean reads were aligned with BTx623 (v3.0.1) reference genome using Burrows-Wheeler Alignment (BWA, v0.7.17)^45^ with default parameters. SNPs were called independently and merged using the Genome Analysis Toolkit (GATK, v4.1.8, HaplotypeCaller)^46^. To compare the EHH decay between the gain- and loss-of-function haplotypes of *Dw7a* and *Dw1*, Extended Haplotype Homozygosity (EHH) of these genes was examined at approximately 500 kbp from each gene, using the rehh package v3.1.2^31^. The integrated Haplotype Scores (iHS) for all the SNPs that were approximately 15 Mb from the genes on chromosomes 7 and 9, were estimated until the EHH score reached a value of 0.05, using the rehh package v3.1.2^32^ according to Yano et al. ^47^. The haplotype network was generated by PopART software v1.7^48^ to visualise of relationships at the population level between individual genotypes.

### Statistical analysis

For analysis of the allelic effects of FD and CL in the F_2_ population, an ANOVA model was followed using Tukey’s HSD test to estimate statistical differences. Different lowercase letters in each plot indicated significant differences in the *post-hoc* test (*P* < 0.05).

## Supplementary Information

Supplementary Information

## Data Availability

All data needed to evaluate the conclusions in the paper are present in the paper and/or the Supplementary Materials.
