# Supplementary material for: The dominance model for heterosis explains culm length genetics in a hybrid sorghum variety
Source: Sci Rep. 2021 Feb 25;11:4532. doi: 10.1038/s41598-021-84020-3 (PMC7907390; doi:10.1038/s41598-021-84020-3)
Supplement: Supplementary file 1 — Supplementary Information [file 41598_2021_84020_MOESM1_ESM.pdf]

Supplementary Information

The dominance model for heterosis explains culm length genetics in a hybrid sorghum variety

Shumpei Hashimoto[1†], Tatsuro Wake[1†], Haruki Nakamura[1], Masaki Minamiyama[1], Satoko Araki-Nakamura[1], Kozue Ohmae-Shinohara[1], Eriko Koketsu[1], Shinnosuke Okamura[1], Kotaro Miura[2], Hideo Kawaguchi[3], Shigemitsu Kasuga[4], Takashi Sazuka[1*]

[1]Bioscience and Biotechnology Center, Nagoya University, Nagoya, Japan.
[2]Faculty of Bioscience and Biotechnology, Fukui Prefectural University, Fukui, Japan.
[3]Graduate School of Science, Technology, and Innovation, Kobe University, Kobe, Japan.
[4]Faculty of Agriculture, Education and Research Center of Alpine Field Science, Shinshu University, Minamiminowa, Japan.

[†]These authors contributed equally to this work.
[*]Corresponding author: sazuka@agr.nagoya-u.ac.jp

Supplementary Figures : S1 to S7
Supplementary Tables : S1 to S3

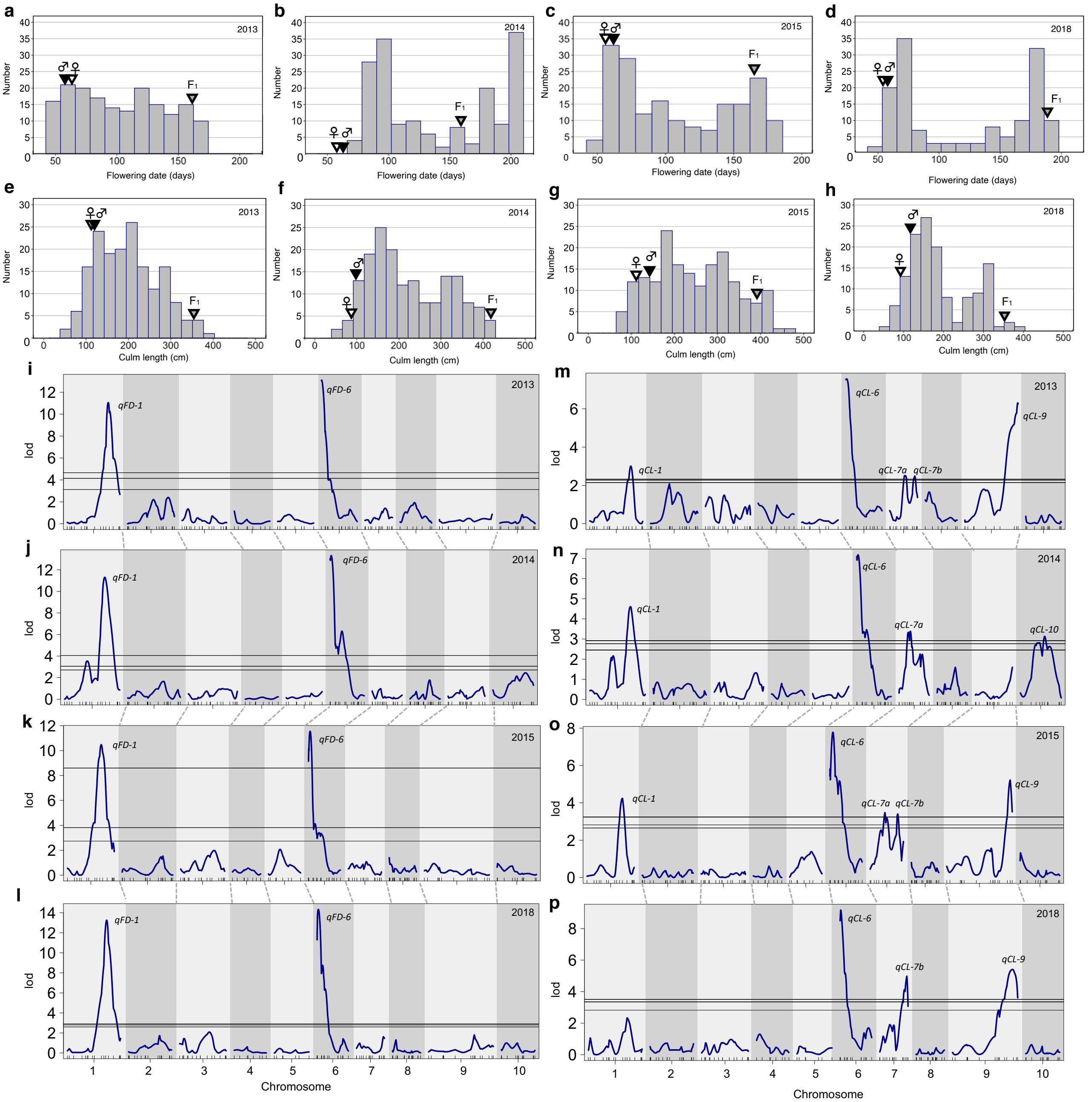

Supplementary Figure S1. Frequency distribution of flowering date (FD) and culm length (CL) in the MS79 × 74LH3213 (F_2) population and QTL analysis results.

a-d) Flowering date (days)**. e-h)** Culm length (cm). The *x*-axis shows the trait value, and the *y*-axis shows the number of plants. The total number of plants; 173, 171, 182, and 144 in 2013, 2014, 2015, and 2018, respectively. An F_2 population from a cross between MS79 and 74LH3213 was analysed for QTLs for FD (**i-l**) and CL (**m-p**). The analysed year is shown in the upper righthand corner of each graph. The numbers and vertical bars on the *x*-axis indicate the chromosome numbers and positions of the DNA markers, respectively. The LOD score is indicated on the *y*-axis. The three horizontal lines in each panel indicate the threshold of the *P* value (0.05, 0.1, and 0.2, incrementally on the y axis). These lines represent the outcomes of 1,000 permutation tests.

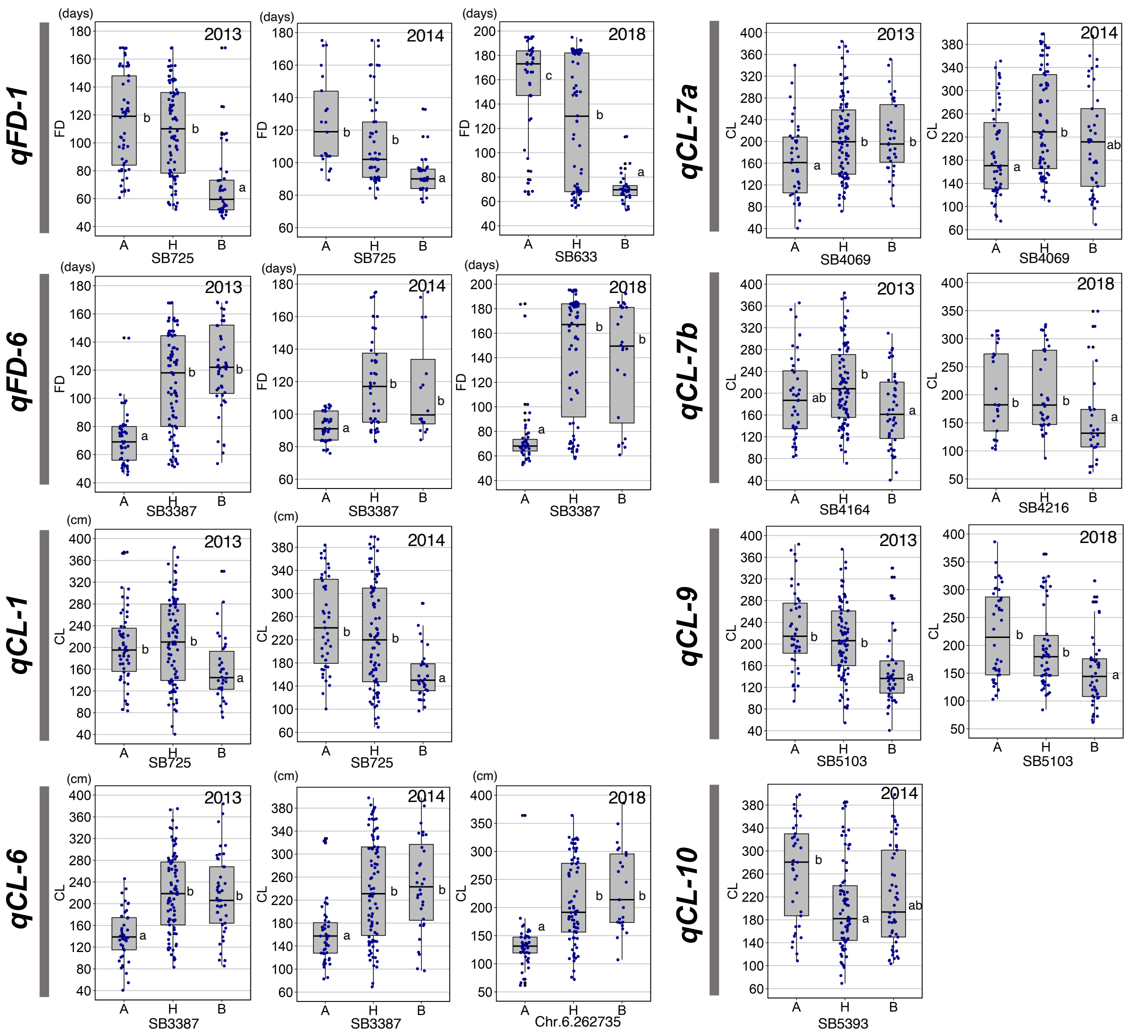

Supplementary Figure S2. Allelic effects of the flowering date and culm length in the five loci.

The allelic effects of *qFD-1* and *qFD-6* on the flowering date and *qCL-6*, *qCL-7a*, *qCL-7b*, and *qCL-9* on the culm length, were evaluated using dot plots. Each point shows an individual plant. Different lowercase letters in each plot indicate significant differences ($P < 0.05$). Data evaluated in 2013, 2014, and 2018 are shown.

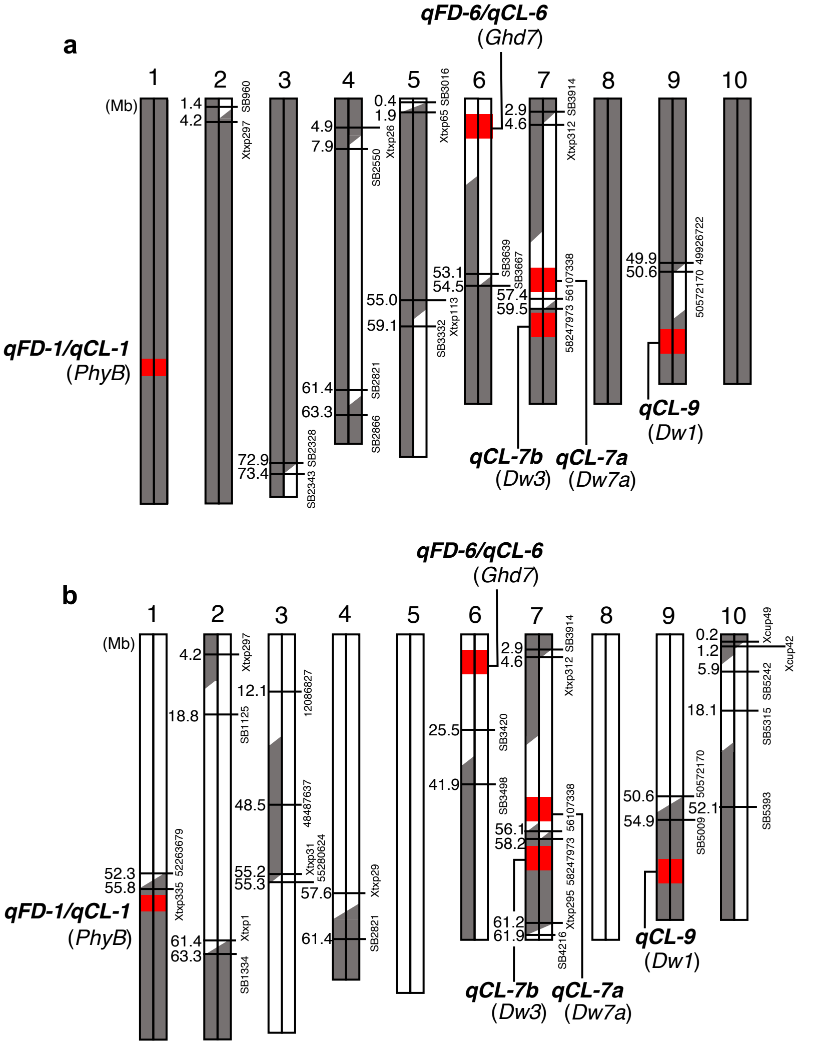

Supplementary Figure S3. Graphical genotype of i5 (BC_1F_3 or BC_1F_4).

Whole genome genotypes of i5 (BC_1F_3 or BC_1F_4) are illustrated. **a**) i5 plants backcrossed with MS79 and **b**) with 74LH3213. The grey and white bars indicate the MS79 and 74LH3213 genomes, respectively, and the red regions indicate the positions of the five identified QTLs in this study (see Table 2). According to the heterozygous loci, homozygous plants were selected by MAS (Marker-Assisted Selection) in the progeny (BC_1F_3 or BC_1F_4; see Figure 3a) to generate the i5 lines. DNA markers and physical positions are indicated on the right and left sides of each chromosome, respectively. These were estimated from the experimental results of the whole-genome genotyping of the parental plants (BC_1F_2), using 144 SSR markers.

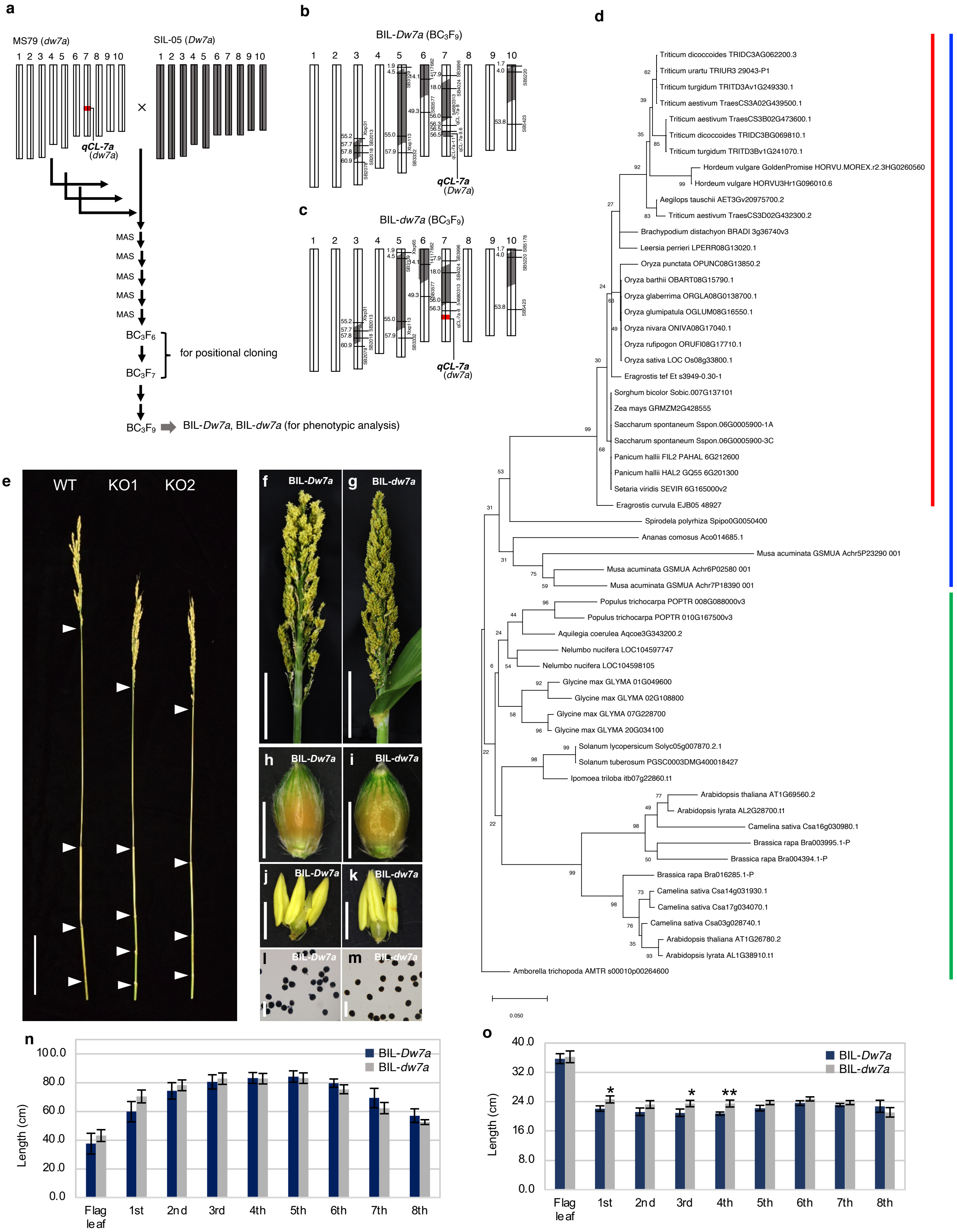
a
MS79 (dw7a)
SIL-05 (Dw7a)
1 2 3 4 5 6 7 8 9 10
1 2 3 4 5 6 7 8 9 10
×
qCL-7a
(dw7a)
MAS
MAS
MAS
MAS
MAS
BC3F6
for positional cloning
BC3F7
BC3F9
BIL-Dw7a, BIL-dw7a (for phenotypic analysis)
b
BIL-Dw7a (BC3F9)
1 2 3 4 5 6 7 8 9 10
qCL-7a
(Dw7a)
c
BIL-dw7a (BC3F9)
1 2 3 4 5 6 7 8 9 10
qCL-7a
(dw7a)
d
Triticum dicoccoides TRIDC3AG062200.3
Triticum urartu TRIUR3 29043-P1
Triticum turgidum TRITD3Av1G249330.1
Triticum aestivum TraesCS3A02G439500.1
Triticum aestivum TraesCS3B02G473600.1
Triticum dicoccoides TRIDC3BG069810.1
Triticum turgidum TRITD3Bv1G241070.1
Hordeum vulgare GoldenPromise HORVU.MOREX.r2.3HG0260560
Hordeum vulgare HORVU3Hr1G096010.6
Aegilops tauschii AET3Gv20975700.2
Triticum aestivum TraesCS3D02G432300.2
Brachypodium distachyon BRADI 3g36740v3
Leersia perrieri LPERR08G13020.1
Oryza punctata OPUNC08G13850.2
Oryza barthii OBART08G15790.1
Oryza glaberrima ORGLA08G0138700.1
Oryza glumipatula OGLUM08G16550.1
Oryza nivara ONIVA08G17040.1
Oryza rufipogon ORUFI08G17710.1
Oryza sativa LOC Os08g33800.1
Eragrostis tef Et s3949-0.30-1
Sorghum bicolor Sobic.007G137101
Zea mays GRMZM2G428555
Saccharum spontaneum Sspon.06G0005900-1A
Saccharum spontaneum Sspon.06G0005900-3C
Panicum hallii FIL2 PAHAL 6G212600
Panicum hallii HAL2 GQ55 6G201300
Setaria viridis SEVIR 6G165000v2
Eragrostis curvula EJB05 48927
Spirodela polyrhiza Spipo0G0050400
Ananas comosus Aco014685.1
Musa acuminata GSMUA Achr5P23290 001
Musa acuminata GSMUA Achr6P02580 001
Musa acuminata GSMUA Achr7P18390 001
Populus trichocarpa POPTR 008G088000v3
Populus trichocarpa POPTR 010G167500v3
Aquilegia coerulea Aqcoe3G343200.2
Nelumbo nucifera LOC104597747
Nelumbo nucifera LOC104598105
Glycine max GLYMA 01G049600
Glycine max GLYMA 02G108800
Glycine max GLYMA 07G228700
Glycine max GLYMA 20G034100
Solanum lycopersicum Solyc05g007870.2.1
Solanum tuberosum PGSC0003DMG400018427
Ipomoea triloba itb07g22860.t1
Arabidopsis thaliana AT1G69560.2
Arabidopsis lyrata AL2G28700.t1
Camelina sativa Csa16g030980.1
Brassica rapa Bra003995.1-P
Brassica rapa Bra004394.1-P
Brassica rapa Bra016285.1-P
Camelina sativa Csa14g031930.1
Camelina sativa Csa17g034070.1
Camelina sativa Csa03g028740.1
Arabidopsis thaliana AT1G26780.2
Arabidopsis lyrata AL1G38910.t1
Amborella trichopoda AMTR s00010p00264600
0.050
e
WT
KO1
KO2
f BIL-Dw7a
g BIL-dw7a
h BIL-Dw7a
i BIL-dw7a
j BIL-Dw7a
k BIL-dw7a
l BIL-Dw7a
m BIL-dw7a
n
BIL-Dw7a
BIL-dw7a
Length (cm)
100.0
80.0
60.0
40.0
20.0
0.0
Flag leaf
1st
2nd
3rd
4th
5th
6th
7th
8th
o
BIL-Dw7a
BIL-dw7a
Length (cm)
40.0
32.0
24.0
16.0
8.0
0.0
Flag leaf
1st
2nd
3rd
4th
5th
6th
7th
8th
*
*
**

Supplementary Figure S4. Phylogenetic and phenotypic analyses of *Dw7a*.

a) Populations that segregated on the *qCL-7a* locus (BC_3F_6 and BC_3F_7) were used for positional cloning, and BIL-*Dw7a* (BC_3F_9) and BIL-*dw7a* (BC_3F_9) were used for phenotypic analysis. **b, c)** Graphical genotypes of BIL-*Dw7a* (**b**) and BIL-*dw7a* (**c**). White and grey bars indicate MS79 and SIL-05 genomes, respectively, and red bars indicate the positions of the *qCL-7a* (*dw7a*). DNA marker names and the physical positions used in this genotyping are indicated on the right and left sides of each chromosome, respectively. **d**) Phylogenetic tree analysis was carried out using the neighbour-joining method based on the amino acid sequence. Numbers at each node indicate bootstrap values (1,000 replicates). The horizontal branch lengths indicate the average substitutions per residue. A red bar indicates a clade of *Poaceae*. Blue and green bars indicate clades of monocot and dicot plants, respectively. **e)** Patterns of internode elongation in the WT and *Dw7a* knock-out (KO) lines. Arrow heads indicate node positions. Scale bar = 10 cm. **f**, **g**) Morphologies of the panicle at the heading stage of *BIL-Dw7a* (**f**) and BIL-*dw7a* (**g**). Scale bars = 50 cm. **h**, **i**) The spiclet of BIL-*Dw7a* (**h**) and BIL-*dw7a* (**i**). Scale bars = 5 mm. **j**, **k**) Phenotypes of the inner floral organs of *BIL-Dw7a* (**j**) and BIL-*dw7a* (**k**). Scale bars = 2 mm. **l**, **m**) Pollen stained by the $KI\text{-}I_2$ of BIL-*Dw7a* (**l**) and BIL-*dw7a* (**m**). Scale bars = 100 µm. **n**, **o**) The lengths of the leaf blade (**n**) and leaf sheath (**o**) were evaluated between BIL-*Dw7a* and BIL-*dw7a* grown under field conditions (two-tailed Student's *t*-test, $*P < 0.05$, $**P < 0.01$, n = 4).

a

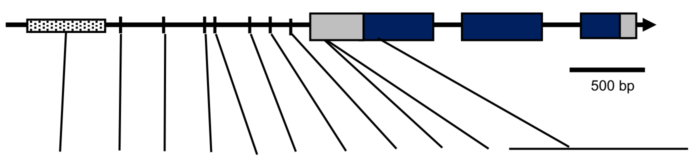

	-2099~-1840	-1516	-1259	-1055	-856	-645	-510	-445	-187	-184	+167	+168	+169	+171
Hap_1 (56)	.	T	A	T	.	.	C	.	C	.	C	A	A	.
Hap_2 (13)	In/Del	G	G	G	+4bp	-8bp	T	+6bp	T	-3bp	T	G	G	+3bp
Hap_3 (13)	In/Del	G	A	T	+12bp	-4bp	T	.	C	.	C	A	A	.
Hap_4 (12)	In/Del	G	A	T	+12bp	.	T	.	C	-14bp	T	G	G	+3bp
Hap_5 (11)	In/Del	G	A	T	C	-8bp	T	.	C	.	T	G	G	+3bp
Hap_6 (10)	In/Del	G	A	T	+12bp	.	T	.	C	-14bp	C	A	A	.
Hap_7 (10)	In/Del	G	A	T	C	.	T	.	C	-10bp	C	A	A	.
Hap_8 (8)	.	G	A	T	+4bp	.	C	.	C	-14bp	T	G	G	+3bp
Hap_9 (6)	In/Del	G	A	T	+12bp	.	T	.	C	-10bp	C	A	A	.
Hap_10 (4)	In/Del	G	A	T	.	-8bp	T	.	C	.	C	A	A	.
Hap_11 (4)	In/Del	G	A	T	+16bp	.	T	.	C	.	C	A	A	.
Others	.	G	A	T	+4bp	.	C	.	C	.	C	A	A	.
	.	G	G	G	+4bp	-8bp	T	+6bp	T	-3bp	T	G	G	+3bp
	In/Del	G	A	T	+16bp	.	T	.	C	-14bp	C	A	A	.
	.	T	A	T	.	.	T	.	C	.	C	A	A	.
	.	G	A	T	+12bp	.	T	.	C	-14bp	T	G	G	+3bp
	In/Del	G	A	T	.	.	T	.	C	-10bp	C	A	A	.
	In/Del	G	G	G	+4bp	-8bp	T	+6bp	T	-3bp	C	A	A	.
	In/Del	G	G	G	+8bp	-8bp	T	.	T	-3bp	T	G	G	+3bp
	In/Del	G	G	T	+4bp	-8bp	T	.	T	-3bp	T	G	G	+3bp
	In/Del	G	A	T	C	-8bp	T	.	C	.	C	A	A	.
	In/Del	G	A	G	+16bp	.	T	.	C	.	C	A	A	.
	.	G	A	T	C	-8bp	T	.	T	-3bp	C	A	A	.
	.	G	A	T	+4bp	-8bp	T	.	T	-3bp	C	A	A	.
	.	G	A	T	+4bp	-8bp	T	.	C	.	C	A	A	.
	.	G	A	T	+12bp	-8bp	T	.	C	.	C	A	A	.
	.	G	A	T	C	-8bp	T	.	C	.	T	G	G	+3bp
	In/Del	T	A	T	C	-8bp	T	.	C	.	T	G	G	+3bp
	In/Del	G	A	T	.	-8bp	T	.	C	.	C	A	A	.
	In/Del	G	A	T	.	-8bp	T	.	T	-3bp	C	A	A	.
	In/Del	G	A	T	.	-8bp	T	.	C	.	T	G	G	+3bp
	In/Del	G	A	T	+12bp	.	T	.	T	-3bp	C	A	A	.
	In/Del	G	A	T	+12bp	.	T	.	C	-10bp	T	G	G	+3bp
	In/Del	G	A	T	+8bp	.	T	.	C	-10bp	C	A	A	.
	In/Del	G	A	T	+12bp	-8bp	C	.	C	.	C	A	A	.
	In/Del	G	A	T	+4bp	-8bp	C	.	C	.	T	G	G	+3bp
	.	G	A	T	C	.	T	.	C	-10bp	C	A	A	.
	.	G	A	T	+4bp	.	T	.	C	-14bp	T	G	G	+3bp
	In/Del	G	A	T	+4bp	.	C	.	C	-14bp	C	A	A	.
	In/Del	G	A	T	+4bp	.	C	.	C	.	C	A	A	.
	.	G	A	T	.	.	T	.	C	-14bp	C	A	A	.
	.	G	A	T	+4bp	.	C	.	C	.	T	G	G	+3bp
	.	G	A	T	+4bp	.	C	.	T	-3bp	T	G	G	+3bp
	.	T	A	T	.	.	T	.	C	-10bp	C	A	A	.
	.	T	A	T	.	.	T	.	C	.	C	A	A	.
	.	T	A	T	.	.	C	.	C	.	T	G	G	+3bp

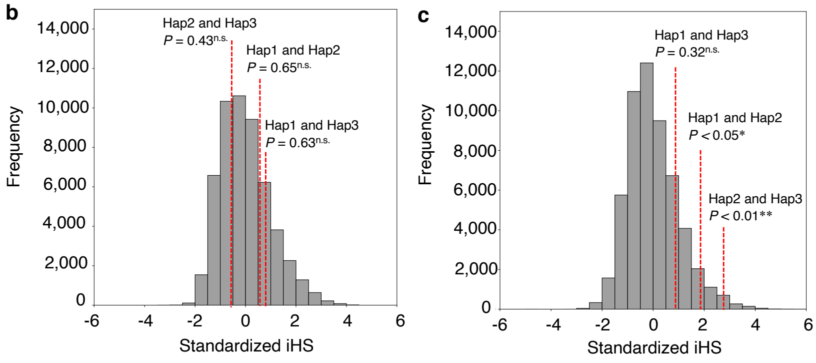

Supplementary Figure S5. Haploid analysis of *Dw7a,* and the distribution of iHS on chromosome 7.

a) Haplotype analysis was carried out using a 2.2 kb upstream initiation codon (including the promoter region) with the coding region among the 187 accessions. Distribution of iHS on chromosomes 7 (**b**) and 9 (**c**). The red vertical lines indicate the position of the iHS for *Dw7a* (**b**) and *Dw1* (**c**), which were estimated using the integrated EHH (Extended Haplotype Homozygosity) of Hap1 and 2, Hap1 and 3, or Hap2 and 3. SNPs in the 15 Mb genomic fragment around the gene were used for the calculation. *P* values are shown near the lines with significance (*;0.05, **;0.01) or with no significance (n.s.).

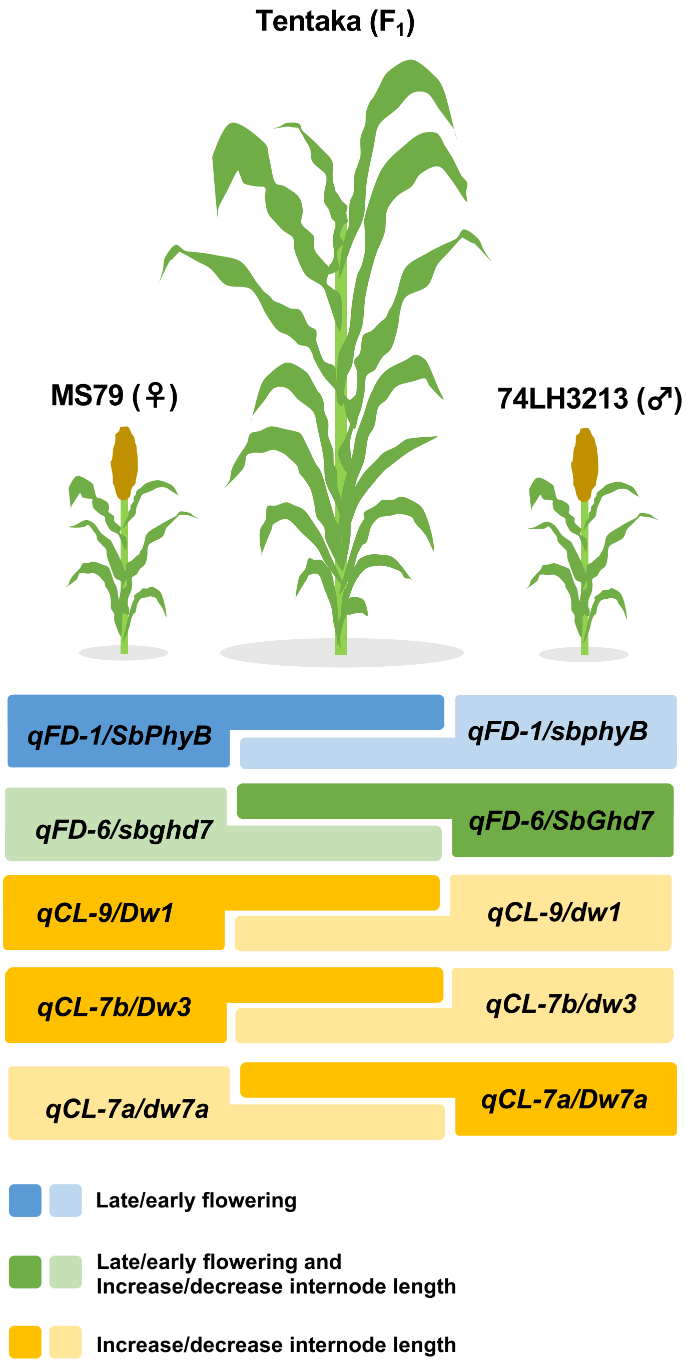

Supplementary Figure S6. Summary of the dominance model for heterosis in 'Tentaka' (F_1 hybrid).

Five genes (*SbPhyB*, *Sbghd7*, *Dw1*, *Dw3*, and *Dw7a*) contributed to the long culm length (CL; See also Table 2). Among the five loci, each recessive allele is loss-of-function resulting in an early flowering date (FD) or short CL. The seed parent (MS79) carries *sbghd7* and *dw7a*, while the pollen parent (74LH3213) carries *sbphyB*, *dw1*, and *dw3*. The F_1 hybrid ('Tentaka') is heterozygous in the five loci, which results in late flowering and a long culm, and this is explained by the dominance model.

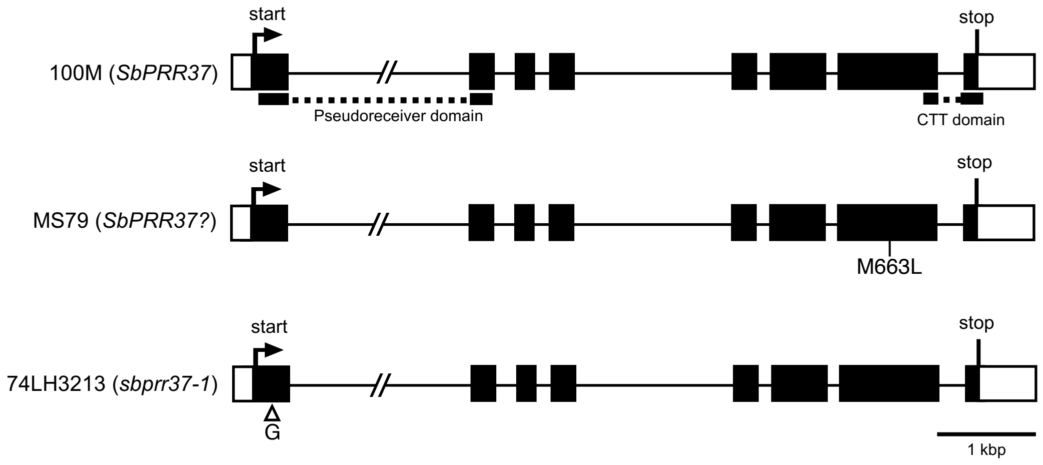

Supplementary Figure S7. Exon-intron structure of *SbPRR37* on MS79 and 74LH3213.

Exons and introns are shown as boxes and black lines, respectively. Black box, protein-coding sequence; white box, untranslated region (UTR). The black bars indicate the functional domains.

Supplementary Table S1. Regional distribution of the Dw7a haplotype of MS79 and 74LH3213.

Dw7a haplotype	Accession	Origin	Region in Fig.6
MS79	IS23514	Ethiopia	Central Africa
	IS23521	Ethiopia	Central Africa
	IS23579	Ethiopia	Central Africa
	IS23590	Ethiopia	Central Africa
	RCV	El Salvador	Central Africa
	E 37	Tanzania	Central Afirica
	143 DINDERAWI 1	Sudan	Central Africa
	E 1091	Sudan	Central Africa
	ZA113 DAWA PAS PARA	Nigeria	Central Africa
	IS18758	Ethiopia	Central Africa
	TX2783	USA	USA
	BATTANBAN	Cambodia	East/South East Asia
74LH3213	IS24939	Zambia	Central Africa
	IS24953	Zambia	Central Africa
	IS24175	Tanzania	Central Africa
	NYAN DOK	Sudan	Central Africa
	FETERITA HARIG 2	Sudan	Central Africa
	IS7310	Nigeria	Central Africa
	IS7679	Nigeria	Central Africa
	AS 4547 JARDIRA	Nigeria	Central Africa
	KA 24	Nigeria	Central Africa
	IS26025	Mali	Central Africa
	IS11473	Ethiopia	Central Africa
	MORABA 74	Ethiopia	Central Africa
	THIBA RED	Ethiopia	Central Africa
	Loussour	Chad	Central Africa
	IS15931	Cameroon	Central Africa
	IS15170	Cameroon	Central Africa
	MN 401	Algeria	Central Africa
	AS 4637 NHORONGO NENPI	Tanzania	Central Afirica
	REDBINE 655	Sudan	Central Africa
	SC 56	Sudan	Central Africa
	ESHOME	South Africa	South Africa
	IS12302	Zimbabwe	South Afirica
	IS29689	Zimbabwe	South Afirica
	AW 70/12 DL/59/1532	South Africa	South Afirica
	E 233 BARNARD RED	South Africa	South Afirica
	RED KAFIR	South Africa	South Afirica
	IS2382	South Africa	South Afirica
	IS24453	South Afirica	South Afirica
	IS27887	South Afirica	South Afirica
	IS29606	South Afirica	South Afirica
	IS19445	Botswana	South Afirica
	IS2838	Zimbabwe	South Africa
	IS19669	Zimbabwe	South Africa
	IS29733	Zimbabwe	South Africa
	RTx432	USA	USA
	B2AR3043	USA	USA
	BTx623	USA	USA
	CAPROCK	USA	USA
	BTx642	USA	USA
	IS473	USA	USA
	IS20816	USA	USA
	IS18712	USA	USA
	IS18729	USA	USA
	RIO	USA	USA
	CHAL WAXY SORGHUM	Korea	East/South East Asia
	LR 399	China	East/South East Asia
	LIAOZA 1	China	East/South East Asia
	SIL-05	Japan	East/South East Asia
	IS32439	India	South Asia
	DHOOTI ANEHULA	India	South Asia
	ALLAKH	Bangladesh	South Asia
	PI 229486 VULGARE	Iran	Middle East
	IS13295	Venezuela	Others
	65I 2786	Mexico	Others
	CRIOLLO CABEZA APRETADA	Honduras	Others

Supplementary Table S2. Primers used in this study.

Marker name	Forward primer (5' -> 3')	Reverse primer (5' -> 3')	Purpose
SB72	CTTCCAGCACTACCTCGTCATGC	AGGAAGCTCTGGATCAGCGTGTT	QTL analysis
Xtxp213	AACATTTCTTCCAGGCTC	GGAAGCAGTGCAGGAT	QTL analysis
SB234	AAAATAAAACCGGCGACAGGACTT	AAGGGCGCCTTTAATTCTGTCAGT	QTL analysis
STSchr.1_11803616	ATGGATATCCTTGGGCAACT	AGCTGCAGTGCCCCCACAAT	QTL analysis
STSchr.1_15571571	CGCCGCGACAAGGGGGTTTA	GTCTACCAAATTCCCCAATT	QTL analysis
SB384	GGCTAGCAGTTAGCTGGCAAGTGT	CTATTGGATGTCAATGGCGAATCA	QTL analysis
SB405	AATGACATTGCCGTAGTGTGCTGT	CATCATACCCTTCGGCTCATCAAT	QTL analysis
SB414	CTATTTTCACGGCTTCCTGCAATC	CGTTGGTCTTGTTCAGGCTCTTG	QTL analysis
SB527	TTTACTGAGTCAGGCCAAACAACG	TTTGCCTATATGTCTCGCCATGTG	QTL analysis
STSchr.1_52263679	ATTCAACGCATGAGGCATG	ACGAAGAAGCTCGAATTTCC	QTL analysis
Xtxp335	TATTTCCTCTTGAAAGAATCAGGG	TATTCATCGAGCAAAAGGCA	QTL analysis
SB622	TCTTTCTCATCATGGCGACTCAAG	AAAAACCTCGTGTGGACGTCAGAT	QTL analysis
SB633	ATGCACTACTGCGCGCTCTACAC	TACAAATGCTAGCACCCTGAGCTG	QTL analysis
SB725	GAATCTGGATTCTGGAGGAGCGTA	TTTTGGACCAACGGAAATAACAGG	QTL analysis
SB791	TCACCACATTGCACAAATCCTTTC	CAGCCTTGGGATACTGCTACTCGT	QTL analysis
SB868	CTCATCAGGCTAAAATGATCACCG	ATCGAGCACCTGAAACATGAAACA	QTL analysis
Xtxp248	GGGTGTCCAATGTTGTCTGC	GGCCGTTACTGTCCCTTACTCA	QTL analysis
SB872	GAGGCGAGAAATTAACCATCGAGA	ACCAACAAGTCTTCGCTCGTAACC	QTL analysis
Xtxp323	TATATGCATGTTTTAGGTCG	CTGTTGTTCCTTTCTTCC	QTL analysis
SB944	AGAAAGAGAGGAAGGGATCAGGGA	GCCTGGTTAACGGAGCTAGCCTAT	QTL analysis
Xtxp96	GCTGATGTCATGTTCCCTCAC	CATTCGTGGACTCTGTCGG	QTL analysis
SB960	GGCATGACATTCTTTCTAGCCCTG	GAAGGAGTCATTGGCCAAATCGTA	QTL analysis
Xtxp297	GACCCATATGTGGTTTAGTCGCAAAG	GCACAATCTTCGCCTAAATCAACAAT	QTL analysis
SB1030	ATCTGCGTCGACCAAGTCAACA	GAGGTTCTACAGGGGCGACATCTA	QTL analysis
SB1125	GCTCATTGCTTAACGAAACTGGTG	CATACAGCCTTGTGCTGGTAGACG	QTL analysis
Xtxp3	AGCAGGCGTTTATGGAAG	ATCCTCATACTGCAGGACC	QTL analysis
SB1176	CGTACGTGCTTCTAGCTCTCGACT	GTTTCAATCATTCATTCAACCGCC	QTL analysis
SB1186	GGCTGATATGATCAAGCTGGTTGT	AGGGATCGAGGCCCTACCTAATTT	QTL analysis
Xtxp298	GCATGTGTCAGATGATCTGGTGA	GCTGTTAGCTTCTTCTAATCGTCGGT	QTL analysis
Xtxp1	TTGGCTTTTGTGGAGCTG	ACCCAGCAGCACTACACTAC	QTL analysis
SB1334	ACTTAGAGGCTACGGAATGGCTCC	AAACTTGGCAGCCACCGATAACTA	QTL analysis
SB1384	GGGAAGCTTGGCTCTTGTTTCATT	GATAGGTGATGAAATGTGGGAGGC	QTL analysis
SB1417	GCAGCAATAGTAGTGGTGGTGTGG	GGGAGAATGAGAAACAGAACGGG	QTL analysis
SB1430	GCTGTATGCTACCTGTTTTCCTCCTG	GCTGAAGAGCATGCACATGAAGAT	QTL analysis
Xtxp7	ACATCTACTACCCTCTCACC	ACACATCGAGACCAGTTG	QTL analysis
SB1554	GTTTTGGCGTCACAAAGCTTATCC	CGAGATTCAGGCCACGTAAACTCT	QTL analysis
SB1562	GAGACGACGCTAATCCATCCAAAC	GCAAGAACACCAGTGCATACATCC	QTL analysis
SB1582	AATGTGCGACAAAGGAGAGGAGAG	GGTTCACCTGAGACGGGTACTAGC	QTL analysis
Xtxp216	GTTCATTTGCCACTCATTC	TATATTGGATAGGCGTGTTG	QTL analysis
SB1749	GGAAAATAGGCTGATGAGGCTGAA	GGGGCAACACAAAGTTACGACTTC	QTL analysis
STSchr.3_6414501	TGAATGACGCATCCATGTCA	AGGGTCCCTTGTGCCCTGGGC	QTL analysis
SB1774	TTGGGAGGTGAGTTAAGCAACCAT	TAGACGACGACCACCCATGATAGA	QTL analysis
Xtxp9	AATAGCACCGCCGCGCG	CATTGTGGAGTCCCTGATAC	QTL analysis
SB1799	GTCACTTGGCATAATCCATCCCAT	CGGGTTATGTCGGTGTATGTAGCA	QTL analysis
STSchr.3_12086827	ACAGACCTCTATCGGTAGAT	TAAATTCTTGGATTTGATGT	QTL analysis
STSchr.3_48487637	TCCAGTTCCAATACTGTTGG	TAGTATCATCAGACATGACAT	QTL analysis
Xtxp31	TGCGAGGCTGCCCTACTAG	TGGACGTACCTATTGGTGC	QTL analysis
STSchr.3_55280624	CTGGTTGCGCCGCAGCCGCC	GTCCACGTCGCTCTTGTGGA	QTL analysis
STSchr.3_58547526	AACAACGCCATGTGATCGTG	CAGTAATGAAGCCGGTCTTCA	QTL analysis
SB2078	CTTAATCAATCACACCTCGCTCCC	TCTTTTCCACACCAAACCATGAGA	QTL analysis
SB2122	GAAGAGCCCAAGGAAGGGAAGATA	AAAAGTGACAGACAACCCGTAGCC	QTL analysis
Xtxp34	TGGTTCGTATCCTTCTCTACAG	CATATACCTCCTCGTCGCTC	QTL analysis
Xtxp70	AGTGACCTTAGCACCAAGCTC	TCAGGTAGCACTAGAG	QTL analysis
Xcup14	TACATCACAGCAGGGACAGG	CTGGAAAGCCGAGCAGTATG	QTL analysis
SB2328	AGCAGCAGTGGTGTCGAGTTACTG	AACGACTCGTGCTGTGTAGGTGTC	QTL analysis
SB2343	GGATCGGAGTGATCCGTAGATAGC	CACAGTACACAACATGCCCATCAA	QTL analysis
Xtxp26	AAGTGTAGTAGCAGTTTAGTCTC	TAGGTATCAAAGGACCAAGG	QTL analysis

SB2550	GATTTCAGTCCCCGTTTGTTTTTG	GAATCATCTCACCACAGCATCACC	QTL analysis
SB2621	ACGTGATCGTGATGGTCTTTTGAA	ATAAAGAATGCCCATTGCTGGTTG	QTL analysis
Xtxp343	CGATTGGACATAAGTGTTC	TATAAACATCAGCAGAGGTG	QTL analysis
Xtxp158	GCCATCAGGGATTGAC	GAGCTTCGCTGAGTAAGG	QTL analysis
Xtxp29	TAGGGCAGTGGTTAGTCGTG	TACAAGTGGTGGTCCGAGG	QTL analysis
SB2821	AATTCCCTGTCGCCCAAACAAC	AAACATCCAGATCCAGGAAGGGTT	QTL analysis
SB2866	GCCTTCGTTCTTCCTCCTGTTCTT	TGGTTTTGTTGATCCTTTTGTCCC	QTL analysis
SB3016	GGAAAAATGAGAAAAGAAACGGGC	GTCTCTGATTGTCAATGCCTCCCT	QTL analysis
Xtxp65	CACGTCGTCACCAACCAA	GTTAAACGAAAGGGAAATGGC	QTL analysis
Xtxp30	AAAAAGGACGCGCAGCTG	CTGGTCTCCACCATCCGTAG	QTL analysis
SB3112	GAGACACGTGAGACGTCCTCCATA	AGGAGCACTACTTCAAGTTTGGCG	QTL analysis
SB3159	GAGCTTGAAGAGGAGCGTCTCG	GTGATCAACCAGGCCGGAAAC	QTL analysis
SB3250	CAAGAAGAGCAAGAAGGGCAAGAA	CAGCTTGAAGGAGTCGGAGAACTG	QTL analysis
Xtxp113	CTCAGCTAATTTAGCCATAG	CAAGTAATAGACGAGTGAAAG	QTL analysis
SB3332	GCAGCTAAACTAAACCCCAGCAGA	ACTCATCGGTCAGTGATCTGTCCA	QTL analysis
Chr.6-74542	TCTTAGGGTCTATTTGGTTCCC	GAATTGCCGATCATCCATGT	QTL analysis
Chr.6-262735	TTGCCAAAGATTTGCAAGC	CATCCTTTGGGTGATCCTACA	QTL analysis
Chr.6-349607	CCAAACGCAAAGCACATAA	GGTGCATCGGTGAAACAATA	QTL analysis
SB3387	CAGAATGGACCATGTGAGTCTTCA	TGTCAAAATCGAACCAAAGGAGGT	QTL analysis
SB3409	CGAAACACCAGATCGAAACAACTG	CGATCCATCGTCTACTCTTCCCAT	QTL analysis
SB3411	GCTCGCATACTAGCTCATCGTTCA	GACTGTCCATGCGATCAGTAGGAA	QTL analysis
SB3420	GAGCCAGCATGCATGATAATTGTT	CACAAAGGCATGACAGTCAATCAA	QTL analysis
Xtxp6	ATCGGATCCGTCAGATC	TCTAGGGAGGTTGCCAC	QTL analysis
SB3481	CCTGAGGATAAGGAATTGCGATGT	TTCTCCTGTGGTGATGAATGGAAA	QTL analysis
SB3498	AGCGGGACTTCTTTTGTGCAATTA	TGTGTATTCATGTGTTGCCTTGTG	QTL analysis
SB3544	TACACAGACGCAGGAACTGAGAGG	CGTCGTAGCATAGTATGTGGCTGC	QTL analysis
SB3577	GAAAAGAAGGTTCCTCCTGCCATT	ACATCCACCCTTCTCTTGGTCAAA	QTL analysis
Xtxp145	GTTCCTCCTGCCATTACT	CTTCCGCACATCCAC	QTL analysis
SB3621	GGTTCTTGGCATTACCTGCAAAAG	GCAATTTGCACTGGTTACTTGCTG	QTL analysis
SB3631	AAAGCCAATCCGGGGAGATGT	CAATCATGGGATCGTACAAGGAGG	QTL analysis
SB3639	CCATCATCGGCACGCAAATCT	GCTGGGAGAGCTAGGAAGAACACA	QTL analysis
SB3667	CAACTCCCATGTGCCATGAAAATA	TATACGTCGCGGGAATCTCTCC	QTL analysis
Xtxp95	TCTCCGTTTGCCCGCCAG	CACCGTACCGCCTCCCGAATC	QTL analysis
SB3913	TTCCCAATCTCCACGCTACTTCTC	TTCTTTGGTCGCTTTCCTTAGCAG	QTL analysis
SB3914	GTCAGTCAGCTTGTTCCTTCCCAT	TACACGAGCAAACAGACGTGTTCA	QTL analysis
Xtxp312	CAGGAAAATACGATCCGTGCCAAGT	GTGAACTATTCGGAAGAAGTTTGGAGGAAA	QTL analysis
SB3963	TTGTAGTTGTGCGTGCCTGAAAAT	CCTGTCTGCACCTCATCTCACACT	QTL analysis
SB3974	TGAACTGAAACCGCTGTCACCTAC	CAGTGCTATATCCGTGCTGCATTC	QTL analysis
SB3996	GAGCTTGTCGAGGAGGTAGTCCAG	TGTCCCCTATTTATACCCCCATCC	QTL analysis
SB4017	GCACGCAGTATTCCACAATGACTC	TTGCACGCTACAAATCAAACTGCT	QTL analysis
SB4069	CACCTACATACGGCAATCCCTGTT	CCCACGTCAAAGATAACTGAACCC	QTL analysis
STS-Chr.7_55114029	TCTCAACATGTTTGCCGTCT	CACCTCATTCATCATCACACA	QTL analysis
STS-Chr.7_56107338	GGACCCTTCAAAATTAGGGA	ACCACAATGCTCAGAACCAA	QTL analysis
STS-Chr.7_58247973	ACTGCCAGACAATATTCCCG	ATGATGATGGTGATGGACCATGT	QTL analysis
SB4143	TCTCCTTTAATTACGACCGACCGA	CTCCATGGATCGACCCTTTTGTT	QTL analysis
SB4164	GCTAGCATACACTTCAATCTGCTTGG	GCCAAACCTAGCTCTTGTCACGAA	QTL analysis
SB4197	CGATCGAGTTTTTCTTGTGGTGTTC	CATGCATCCATGTTCGTCTTCTCT	QTL analysis
Xtxp295	AAATCATGCATCCATGTTCGTCTTC	CTCCCGCTACAAGAGTACATTCATAGCTTA	QTL analysis
SB4216	CACTCTCTACCACCACTGGCCTTT	AGATTTGGACTTGTGCCACTCCTC	QTL analysis
SB4288	TCACAGCTTTGAACTGTGTGTTGC	CAAAACTTGTCCAAGCCATCACAT	QTL analysis
STSchr.8_629127	TGGGATTAGGGGACAACATT	TCCCCATCTCACCACTTCATA	QTL analysis
STSchr.8_893977	GACATTGGAACACTGCATGA	CAGGCAATAGCAAGGCATGTA	QTL analysis
STSchr.8_2064712	CCTGGAACTGGTACGCGGTGGG	CAATCCCATCACCACCTAGGCCA	QTL analysis
STSchr.8_2387593	AGTGCAAACAACGAGATTGG	TTAGGGGGCAAATGGAAGAT	QTL analysis
STSchr.8_2961744	CTGCAGCACACAATGCTTAAA	TTGTTTATCTCTGCATCGCC	QTL analysis
SB4341	CTCCTGCCTCCCTTGCTAGTTTTC	TTAGCAGATTTCTTGGGAGAACGG	QTL analysis
STSchr.8_3167823	TTGAATTCTTGAGAGCAGCG	AGATGTCGACGCATTAGACAAAT	QTL analysis
SB4386	TTCGTTTGCTACCTCACACAGCTT	CTCTGGTGACGATGGTTTTGATTG	QTL analysis
STSchr.8_4797082	GGGTTTCCAGCAATGAATATG	TTACGAGCCGCACTCAATAA	QTL analysis

STSchr.8_12239241	CGGCACACAGTAACAATCCA	TGGCTGCTGCTCCGTGGCCG	QTL analysis
STSchr.8_14677562	ATTCATCTCCGTTGGCTGCC	TTATAAGAGACCAGTTTGG	QTL analysis
STSchr.8_16017038	CTTGTGTCCAACAAGCAACCTA	AACAACACCCAAAATACAGACC	QTL analysis
STSchr.8_17072732	GGTGATTTGTTGGGTAGTTG	TTTTATTGAGTTAGATCGT	QTL analysis
SB4513	CGGTTCCTTCAACAAATCTCATCA	CTGTTTGGATTCAGACGGGTCAT	QTL analysis
STSchr.8_47348158	ACCGCAGCATGATAAGGTCT	CATCGTTATCTGACGAGACGAA	QTL analysis
STSchr.8_47517547	TTCCAGTTCAGTCCATTTCCA	TCGGCTTATGTTGAACTCAGA	QTL analysis
STSchr.8_48168003	TTCATCGATCCTTCCTTTCC	TGCTACAATTGCTCCCATCA	QTL analysis
Xtxp18	ACTGTCTAGAACAAGCTGCG	TTGCTCTAGCTAGGCATTTC	QTL analysis
Xtxp321	TAACCCAAGCCTGAGCATAAGA	CCCATTCACACATGAGACGAG	QTL analysis
SB4579	CACAACTCACTACTGGCACAACGA	CGCTAGATCCAATTCAGCTGTGTG	QTL analysis
Xtxp289	AAGTGGGGTGAAGAGATA	CTGCCTTTCCGACTC	QTL analysis
SB4688	CTGTAAGCATGATGAAGGTCGTGG	AAGAAGGTGATGACAGGGATGGAG	QTL analysis
SB4725	AACCCCACCGTAACATCAGTCAGT	AATGGCAGCTACCACTCACTCCTC	QTL analysis
Xtxp258	CACCAAGTGTCGCGAACTGAA	GCTTAGTGTGAGCGCTGACCAG	QTL analysis
SB4780	TTGAGTTGCTTCTAAATCGCCCAT	TGACGGCCCTGTTCATAAAGAAAT	QTL analysis
STSchr.9_4353198	CCTTCCCAACCAATCTGTAA	TGAAACTTCCTGTTTCAGCCT	QTL analysis
STSchr.9_5342304	GTGTCACCTTGAAGATTGTGTC	TATGGTATAACGGTGCCAAGTG	QTL analysis
SB4-15	GCTGCTAAGCCGTGCTGA	TTATTTGGGTGAAGTAGAGGTGAACA	QTL analysis
STSchr.9_9153442	CTTTGCTTCTGCGGAGTATT	TTGACAGGCATAGCAGTTTCTG	QTL analysis
STSchr.9_39783834	CCATTTTGGCCATCACAAG	TCTCTCACGAACATGGTCAA	QTL analysis
SB4894	ACGCGTACACTCCTTCAGTCCTTC	CAAGCGTTCAGCAACAGTGAAACT	QTL analysis
STSchr.9_49926722	GTGGAAAAGCTCTACCTCCA	GTCAGAGAGACCGACCAAGA	QTL analysis
STSchr.9_50572170	TCACTGATGATGACCAGCGA	TTCTCCCGTGATCACCACGT	QTL analysis
SB5009	CTTCAGAGTCAGAGGTTGCTGCTG	CTGTTTGTCCTCTTCCACGCTGTT	QTL analysis
Xtxp107	CAAAGTGAGCGTGGTC	GGACAGGGATAACATAACATA	QTL analysis
SB5103	CCCCATAAATCTGTGCAAAACACA	CATGAATTCACATGATTTTCCCTCC	QTL analysis
Xcup49	TCCACCTCCATCATCTTTCC	CTCCACCACCTCCATGACTC	QTL analysis
Xcup42	CACACCTGTCTCTCTTCTCCG	AGATCATCTTCGCCTTCCTC	QTL analysis
SB5242	GCTACAAACATCTGAGACTGCCGA	TCAGAGGACCCTGTTTCGTTTGAT	QTL analysis
SB5284	GATGTGTTTGCAAGAACAGCTTAAC	GTTGGTCCAAAAACAAGCCCTGTA	QTL analysis
SB5293	TGAATAATGCACGCAGTAGCGTCT	TATTCCCACGGCTCGCTAGCTACT	QTL analysis
SB5315	TCACTACCAAATGCACGAAGGAAA	GTGATAGGCTCATCCCCTGTAACG	QTL analysis
SB5393	CATCGAAACGGAAGACACAAAACA	CCTACAGTGTGTCTGGCTGCAAGT	QTL analysis
SB5407	GAGTCTGCGCTTATTGTGCCTTTT	TGCCTTTTTGCCAGATCTTTCTTC	QTL analysis
SB5417	TGTTCCTACAGCTCCAAAGCTTCC	GCACCAGGCAATGGATACAATACA	QTL analysis
Xtxp141	TGTATGGCCTAGCTTATCT	CAACAAGCCAACCTAAA	QTL analysis
SB5529	CAGCAAATTTCAGGTGGTTTTTGAC	ATTATATCTCTGCGTGCAACGGCT	QTL analysis
SB5556	AGCTTCAAAGATGGCCAAAACAGA	GCAGAGCCACAAGTCAAGGTGTTA	QTL analysis
Chr.7-51692736	ACGCATAGGCACTCCAATCT	GGTGTTCGCTTGGGTCTTTG	fine mapping
Chr.7-52193290	CCCTTTGGTTCCACCAATTA	TGGGAGCAGCCTTTAATGA	fine mapping
Chr.7-52591220	CCGCTTGAGTTAGTCCATTG	TTGTTGTCTGGTTTGCTTGC	fine mapping
SSR2-4069	CACCTACATACGGCAATCCCTGTT	CCCACGTCAAAGATAACTGAACCC	fine mapping
Chr.7-53383271	GTGACAATCATGTGCAAGTGTC	CCATATGGGCTCAAACAATTC	fine mapping
Chr.7-53430275	TGTTTAAAGCAGTCAGGGGT	AAGGCAACTGGTTGGTGGGAT	fine mapping
Chr.7-53896547	TCTCTCGGCTCTATCAGCGC	ATTATGTCGGGTGCCAAAGA	fine mapping
Chr.7-54680313	CAGAGCCGCTTGTTTGGTTTA	GCTTTTGTACGGCTTTGGGT	fine mapping
qCL-7a-8	ACTTTCTGCAAAATAGATCT	TTAGAACCCGACAAAAAAGA	fine mapping
Chr.7-55081257	ATAGCAGCAGACCCACATCTTT	TCGTAGTTTGTGTGAGTGGTGA	fine mapping
Chr.7-55114030	TTGTCTCAACATGTTTGCCG	CACCTCATTCATCATCACACA	fine mapping
Chr.7-56123867	CGTTTTTCTTACTGTGACCCG	ATTGTTTGAGGTTCCGGTTG	fine mapping
qCL-7a-9.6	TCAGCCGCCAATAGTATTTC	TGATGCGTAGTATATGGTTC	fine mapping
qCL-7a-9	ACTACGTGCTGATGACCATC	TCACAGGCGGTCGGGTAAGC	fine mapping
qCL-7a-9.8	TAAATCCGATCCCAAATGATC	AGCTACCTTTGCCATATGAG	fine mapping
qCL-7a-9.9	TCAAAAGATTTACAAATTGTG	TTCAAATGTTTAATATGATTAG	fine mapping
qCL-7a-10m	AAGGCCTTACTATAACTTTG	TACAGTACATACTTCCTCAC	fine mapping
qCL-7a-11	GGCATTAAGCCATGGCCATG	ACAAATCAATGAGCAACAAA	fine mapping
Chr.7-56262843	TCCACGTCGCCGAACGTGA	TTGCACGTAGCTCCAATCCA	fine mapping
Chr.7-56574306	TTGTTTGGCATTTGCAGTG	GATGAAGAACAGAGGCTTTCCT	fine mapping
Chr.7-56603489	TCGACTCCCGAGAGATTAACA	AGAAGGAGGAGTACTTGCGGA	fine mapping
Dw7a-qRT	TCCCTTCTGCTTGTTCCCTG	GCTGTCTTGTGGTCCCTTCA	qRT-PCR
SbUbi-qRT	GGTTCGGGAGGTGGCCTAGGT	AGCATGTACATTCCCAGCGGTA	qRT-PCR
Os7a_Crispr_oligo_1	AAACGCTGGTTGAACCACCTCAGC		knock-out
Os7a_Crispr_oligo_2	GTTGGCTGAGGTGGTTCAACCAGC		knock-out
Os7a_check1	CGCCAAGCTCCGCGAGCTCGTC	CAGTAGTGGCGGAAGGTGAAGG	knock-out

Supplementary Table S3. Sorghum accessions examined in this study.

Accession / variety	Origin	Region	*Dw7a* Haplotype
MN 401	Algeria	Central Africa	Hap 1
IS15931	Cameroon	Central Africa	Hap 1
IS15170	Cameroon	Central Africa	Hap 1
Loussour	Chad	Central Africa	Hap 1
IS11473	Ethiopia	Central Africa	Hap 1
MORABA 74	Ethiopia	Central Africa	Hap 1
THIBA RED	Ethiopia	Central Africa	Hap 1
NYAN DOK	Kenya	Central Africa	Hap 1
IS26025	Mali	Central Africa	Hap 1
IS7310	Nigeria	Central Africa	Hap 1
IS7679	Nigeria	Central Africa	Hap 1
AS 4547 JARDIRA	Nigeria	Central Africa	Hap 1
KA 24	Nigeria	Central Africa	Hap 1
REDBINE 655	Sudan	Central Africa	Hap 1
FETERITA HARIG 2	Sudan	Central Africa	Hap 1
SC 56	Sudan	Central Africa	Hap 1
IS24175	United Republic of Tanzania	Central Africa	Hap 1
AS 4637 NHORONGO NENPI	United Republic of Tanzania	Central Africa	Hap 1
IS24939	Zambia	Central Africa	Hap 1
IS24953	Zambia	Central Africa	Hap 1
LR 399	China	East/South East Asia	Hap 1
LIAOZA 1	China	East/South East Asia	Hap 1
SIL-05	Japan	East/South East Asia	Hap 1
74LH3213	Japan	East/South East Asia	Hap 1
CHAL WAXY SORGHUM	South Korea/North Korea	East/South East Asia	Hap 1
PI 229486 VULGARE	Iran	Middle East	Hap 1
IS19445	Botswana	South Africa	Hap 1
IS24453	South Africa	South Africa	Hap 1
IS27887	South Africa	South Africa	Hap 1
IS29606	South Africa	South Africa	Hap 1
IS2382	South Africa	South Africa	Hap 1
ESHOME	South Africa	South Africa	Hap 1
AW 70/12 DL/59/1532	South Africa	South Africa	Hap 1
E 233 BARNARD RED	South Africa	South Africa	Hap 1
RED KAFIR	South Africa	South Africa	Hap 1
IS12302	Zimbabwe	South Africa	Hap 1
IS29689	Zimbabwe	South Africa	Hap 1
IS2838	Zimbabwe	South Africa	Hap 1
IS19669	Zimbabwe	South Africa	Hap 1
IS29733	Zimbabwe	South Africa	Hap 1
ALLAKH	Bangladesh	South Asia	Hap 1
IS32439	India	South Asia	Hap 1
DHOOTI ANEHULA	India	South Asia	Hap 1
IS473	USA	USA	Hap 1
IS20816	USA	USA	Hap 1
RTx432	USA	USA	Hap 1
B2AR3043	USA	USA	Hap 1
BTx623	USA	USA	Hap 1
CAPROCK	USA	USA	Hap 1
BTx642	USA	USA	Hap 1
RIO	USA	USA	Hap 1
IS18712	USA	USA	Hap 1
IS18729	USA	USA	Hap 1
CRIOLLO CABEZA APRETADA	Honduras	Others	Hap 1
65I 2786	Mexico	Others	Hap 1
IS13295	Venezuela	Others	Hap 1
IS23514	Ethiopia	Central Africa	Hap 2
IS23521	Ethiopia	Central Africa	Hap 2
IS23579	Ethiopia	Central Africa	Hap 2
IS23590	Ethiopia	Central Africa	Hap 2

IS18758	Ethiopia	Central Africa	Hap 2
ZA113 DAWA PAS PARA	Nigeria	Central Africa	Hap 2
143 DINDERAWI 1	Sudan	Central Africa	Hap 2
E 1091	Sudan	Central Africa	Hap 2
E 37	United Republic of Tanzania	Central Africa	Hap 2
BATTANBAN	Cambodia	East/South East Asia	Hap 2
TX2783	USA	USA	Hap 2
RCV	El Salvador	Others	Hap 2
MS79A	unknown	unknown	Hap 2
IS14290	Botswana	South Africa	Hap 3
IS29392	Lesotho	South Africa	Hap 3
NYAKASOBA BEST	Lesotho	South Africa	Hap 3
IS13868	South Africa	South Africa	Hap 3
IS13782	South Africa	South Africa	Hap 3
IS26737	South Africa	South Africa	Hap 3
EAR FROM PIETESBURG DL/60/107	South Africa	South Africa	Hap 3
S. BASUTORUM DL/60/97	South Africa	South Africa	Hap 3
IS29233	Swaziland	South Africa	Hap 3
IS29241	Swaziland	South Africa	Hap 3
IS29314	Swaziland	South Africa	Hap 3
CAPE COLO 28/53	Zimbabwe	South Africa	Hap 3
COL/PAK/1991/IBPGR/2724(2)	Pakistan	South Asia	Hap 3
SC 426	Japan	East/South East Asia	Hap 4
8493	Turkey	Middle East	Hap 4
KOCADARI	Turkey	Middle East	Hap 4
IS12804	Turkey	Middle East	Hap 4
IS4060	India	South Asia	Hap 4
IS4372	India	South Asia	Hap 4
IS5919	India	South Asia	Hap 4
IS5295	India	South Asia	Hap 4
EC 18868	Nepal	South Asia	Hap 4
IS13122	Pakistan	South Asia	Hap 4
SAP-155	Brazil	Others	Hap 4
IS14131	Portugal	Others	Hap 4
IS1219	China	East/South East Asia	Hap 5
IS30443	China	East/South East Asia	Hap 5
IS30451	China	East/South East Asia	Hap 5
IS30460	China	East/South East Asia	Hap 5
BIG WHITE HULL	China	East/South East Asia	Hap 5
TAKAKIMI	Japan	East/South East Asia	Hap 5
IS30533	South Korea/North Korea	East/South East Asia	Hap 5
KOUSHUU ZAIRAISHU	South Korea/North Korea	East/South East Asia	Hap 5
72-8-13	Taiwan	East/South East Asia	Hap 5
IS31706	Yemen	Middle East	Hap 5
IS35803	Russia	Others	Hap 5
IS11026	Ethiopia	Central Africa	Hap 6
IS18551	Ethiopia	Central Africa	Hap 6
PHATSAI	Morocco	Central Africa	Hap 6
IS2205	India	South Africa	Hap 6
IS3971	India	South Asia	Hap 6
IS4581	India	South Asia	Hap 6
IS18039	India	South Asia	Hap 6
KALJANPUR	India	South Asia	Hap 6
COL/PAK/1989/IBPGR/2444(1)	Pakistan	South Asia	Hap 6
COL/PAK/1989/IBPGR/2411(1)	Pakistan	South Asia	Hap 6
IS14154	Uganda	Central Africa	Hap 7
HONGKER	China	East/South East Asia	Hap 7
KIKUCHI ZAIRAI	Japan	East/South East Asia	Hap 7
OOTOYO-MURA ZAIRAI	Japan	East/South East Asia	Hap 7
IKEDACHO MATSUO ZAIRAI	Japan	East/South East Asia	Hap 7
IS8012	Japan	East/South East Asia	Hap 7
HIMEKI ZAIRAI	Japan	East/South East Asia	Hap 7

PI 220636 Q 2/3/56	Afghanistan	South Asia	Hap 7
IS27805	Hungary	Others	Hap 7
IS27806	Hungary	Others	Hap 7
E 9	Chad	Central Africa	Hap 8
HEGARI MALOWAR	Sudan	Central Africa	Hap 8
AKAHO	Japan	East/South East Asia	Hap 8
TOKIBI	Japan	East/South East Asia	Hap 8
KANAGAWAZAIRAI	Japan	East/South East Asia	Hap 8
IS31714	Yemen	Middle East	Hap 8
WAD YABOO 132/53	Zimbabwe	South Africa	Hap 8
IS21599	unknown	unknown	Hap 8
IS12937	Ethiopia	Central Africa	Hap 9
IS11619	Ethiopia	Central Africa	Hap 9
IS22616	Myanmar	East/South East Asia	Hap 9
IS6421	India	South Asia	Hap 9
IS5301	India	South Asia	Hap 9
IS20740	USA	USA	Hap 9
IS33353	Kenya	Central Africa	Hap 10
NYIRARUMOGO	Rwanda	Central Africa	Hap 10
IS25548	Rwanda	Central Africa	Hap 10
IS22609	Sri Lanka	South Asia	Hap 10
IS10757	Chad	Central Africa	Hap 11
IS3547	Sudan	Central Africa	Hap 11
IS20956	Indonesia	East/South East Asia	Hap 11
IS10302	Thailand	East/South East Asia	Hap 11
IS 11034	Ethiopia	Central Africa	Others
IS22799	Somalia	Central Africa	Others
MILO PET. 139/51 EX TANGANYIKA	Central Africa	Central Africa	Others
IS 8267	United Republic of Tanzania	Central Africa	Others
IS4631	India	South Asia	Others
IS33844	India	South Asia	Others
IS23586	Ethiopia	Central Africa	Others
RTx430	USA	USA	Others
SC NO.0217 CI1197	India	South Asia	Others
SOR 1	Romania	Others	Others
IS31681	Algeria	Central Africa	Others
IS16151	Cameroon	Central Africa	Others
AKLMOI WHITE	Kenya	Central Africa	Others
SCHROCK	Morocco	Central Africa	Others
GULUM ABIAD	Sudan	Central Africa	Others
AGIRA	Sudan	Central Africa	Others
MUGBASH WHITE	Sudan	Central Africa	Others
MENDO	Sudan	Central Africa	Others
ZIRA-EL-SABI	Sudan	Central Africa	Others
E 276 FRAMIDA	Uganda	Central Africa	Others
IS30417	China	East/South East Asia	Others
NUO CAO LIANG	China	East/South East Asia	Others
KOUCHI OUKAWA ZAIRAI	Japan	East/South East Asia	Others
AS 5781 HUAN SA PHAUNG AH LPYSU	Myanmar	East/South East Asia	Others
MOCTAC LOCAL	South Korea/North Korea	East/South East Asia	Others
CHOONCHAN LOCAL	South Korea/North Korea	East/South East Asia	Others
15065	Saudi Arabia	Middle East	Others
IS23992	Yemen	Middle East	Others
IS28451	Yemen	Middle East	Others
MAKHOTLONG I	Lesotho	South Africa	Others
IS24503	South Africa	South Africa	Others
IS29269	Swaziland	South Africa	Others
MARIANGARIJORA MUDDAHIHAL	India	South Asia	Others
Juar	India	South Asia	Others
87-9-21-3-1	Pakistan	South Asia	Others
COL/PAK/1989/IBPGR/2427(5)	Pakistan	South Asia	Others
COL/PAK/1989/IBPGR/2439(1)	Pakistan	South Asia	Others
IS12697	Australia	Others	Others
A-6129	Former Soviet Union	Others	Others
IS12945	Nicaragua	Others	Others
